# Clarifying mechanisms and kinetics of programmable catalysis

**DOI:** 10.1016/j.isci.2024.109543

**Published:** 2024-03-20

**Authors:** Brandon L. Foley, Neil K. Razdan

**Affiliations:** 1 Lawrence Livermore National Laboratory, 7000 East Avenue, Livermore, CA 94550, USA; 2 Department of Chemistry, Massachusetts Institute of Technology, Cambridge, MA 02139, USA

**Keywords:** Chemistry, Catalysis, Mathematical physics

## Abstract

Programmable catalysis—the purposeful oscillation of catalytic potential energy surfaces (PES)—has emerged as a promising method for the acceleration of catalyzed reaction rates. However, theoretical study of programmable catalysis has been limited by onerous computational demands of integrating the stiff differential equations that describe periodic cycling between PESs. This work details methods that reduce the computational cost of finding the limit cycle by ≳10^8^×. These methods produce closed-form analytical solutions for didactic case studies, examination of which provides physical insights of programmable catalysis mechanisms. Generalization of these analyses to more complex reaction networks, including CO oxidation on Pt (111) surfaces, exposes the key catalyst properties required to achieve enhanced rates and conversions via one of two programmable catalysis mechanisms: quasi-static (high frequency) and stepwise (intermediate frequency). Analytical description of each mechanism is critical in understanding the consequences of the Sabatier principle on achievable rate enhancement through programmed catalysis.

## Highlights

• Programmed catalysis rate is described by Langmuir-Hinshelwood rate functions
• Steps in catalytic programs are optimized to subsections of the catalytic cycle
• The Sabatier principle imposes limits on maximum rate in programmed catalysis
• Relationships between scaling relations and programmed mechanism are identified

## Graphical abstract

**Figure:**
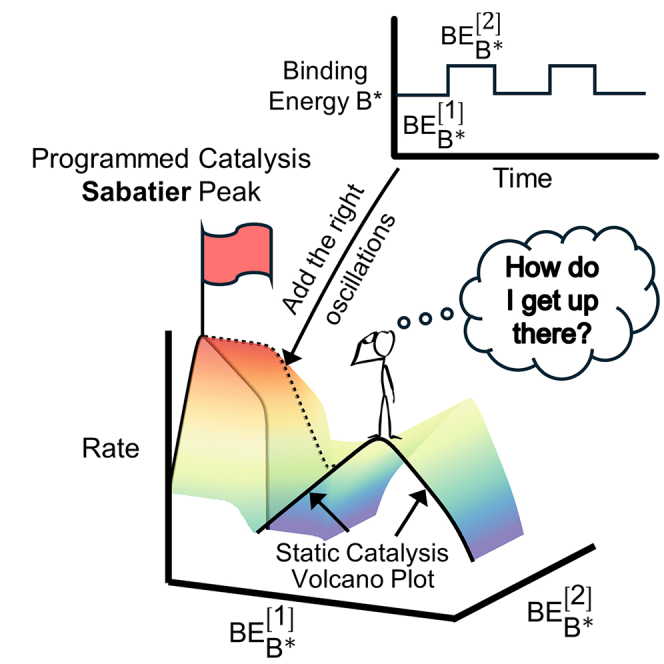

## Introduction

Experimental^1^^,^^2^^,^^3^^,^^4^^,^^5^^,^^6^^,^^7^^,^^8^^,^^9^^,^^10^ and theoretical^11^^,^^12^^,^^13^^,^^14^^,^^15^^,^^16^^,^^17^^,^^18^ reports have demonstrated that purposeful periodic input of thermodynamic work (e.g., by oscillation of applied electrode potential) can effect orders-of-magnitude improvement in catalytic turnover rates and overcome static equilibrium limits to chemical conversion akin to molecular motors/ratchets in biological systems.^19^^,^^20^ These strategies attenuate both kinetic and thermodynamic barriers by leveraging the kinetic asymmetry of two or more energetic states of the catalytic material. For example, an effective forcing program may accelerate rate by deliberately oscillating between two distinct catalytic conditions (e.g., two applied electrode potentials) which, respectively, promote reactant adsorption and product desorption. Oscillating between these two energetic states of the catalyst at frequencies commensurate with elementary step rate constants proffers a method to achieve time-averaged turnover rates exceeding the static maximum prescribed by the Sabatier principle. While this work focuses on forced changes to reaction energies by controlled stimuli, passive forcing—where the effective switching between potential energy surfaces is spontaneous and dictated entirely by innate kinetics of chemical transformation and catalyst restructuring—has also been shown to influence catalytic reaction rates.^21^^,^^22^

The virtue of inducing catalytic oscillations through external stimuli has recently been demonstrated by measurement and calculation of rates,^12^^,^^13^^,^^14^^,^^15^^,^^23^^,^^24^^,^^25^ conversions,^16^^,^^26^^,^^27^^,^^28^ and selectivities^29^^,^^30^ of various catalytic systems at the limit cycle. However, general theoretical understanding of programmed catalysis has been impeded by the vastness of variable spaces describing intrinsic reaction kinetics and forcing waveforms. These challenges are exacerbated by the lack of closed-form solutions and slow computation of limit cycles for programmed catalysis, with forward integration approaches taking up to several days to solve the stiff, coupled ordinary differential equations (ODEs) for a single reaction condition.^31^

In this work, we detail strategies for the calculation and optimization of programmed limit cycles disencumbered from forward integration of ODEs—the numerical solutions for which do not provide the mechanistic clarity of closed-form rate expressions. Our developments provide analytical expressions for reaction rate during programmable catalysis that expose the underpinnings of rate promotion through previously termed “catalytic resonance” mechanisms. In this context, resonance is common parlance for describing the finite frequency band wherein maximal rate enhancement is observed. This terminology, however, is dissonant with the underlying mechanism because the catalytic system does not generally have a natural frequency for oscillations to resonate with. Indeed, this incongruence between terminology and mechanism has been recognized by comparison of characteristic timescales of catalysis with rigorous criterion for resonance.^16^^,^^32^^,^^33^^,^^34^^,^^35^ For these reasons, in this work we refer to catalytic resonance as “stepwise programmed catalysis.” This terminology is chosen to capture two salient features specific to this mechanism of programmed rate promotion: (i) that each instruction in the catalytic program executes a distinct segment of the overall reaction, and (ii) that the order of instructions in the catalytic program is important (i.e., the instructions in the catalytic program do *not* commute). In addition to clarifying these mechanistic details of stepwise catalysis, we explore rate promotion under high-frequency oscillations where characteristic timescales of reaction exceed those of the forcing program.^35^^,^^36^^,^^37^ In these “quasi-static” mechanisms, surface coverages become effectively time-invariant, or quasi-static, throughout the duration of each oscillation cycle. Under these conditions, only the set of program instructions are important; the order of instructions in the catalytic program is irrelevant.

From these learnings, we establish general design principles that dictate the disparate requirements for programmed rate enhancement by stepwise or quasi-static mechanisms in the context of linear free energy (LFE) scaling relationships. Specifically, we show that, based on the magnitude and polarity of LFE scaling parameters, catalytic reactions can be either (i) promotable by stepwise programs, (ii) promotable by quasi-stasis, or (iii) not promotable by programmed catalysis. The classification of catalytic reactions into these categories is made based on closed-form kinetic and thermodynamic criteria that describe reaction rate, surface coverage, and limits to supra-equilibrium conversion.

Improvements in computational efficiency also enable facile discovery of optimal waveforms for enhanced reaction rates. These globally optimized catalytic programs achieve reaction rates that are orders-of-magnitude larger than previously reported local optima. Extension of these learnings to catalytic CO oxidation on Pt (111) facets reveals that periodic straining from compressed (−5% strain) to stretched (+5% strain) surfaces accelerates rate ∼20-fold relative to the Sabatier maximum. Moreover, rates of programmed catalysis are quantitatively predicted through simple rate expressions that capture changes in rate-controlling steps and surface coverage as a function of oscillation frequency and lattice strain.

## Results and discussion

### Finding analytical solutions for limit cycles in dynamic catalysis

**Scheme 1:**
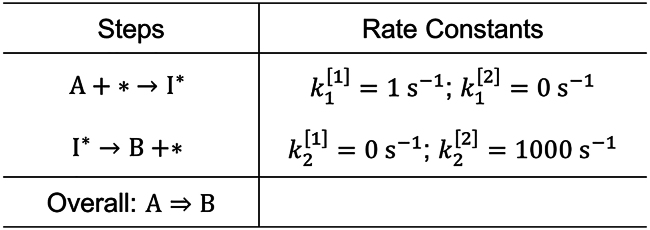
Simplest programmable catalysis reaction network

**Figure 1:**
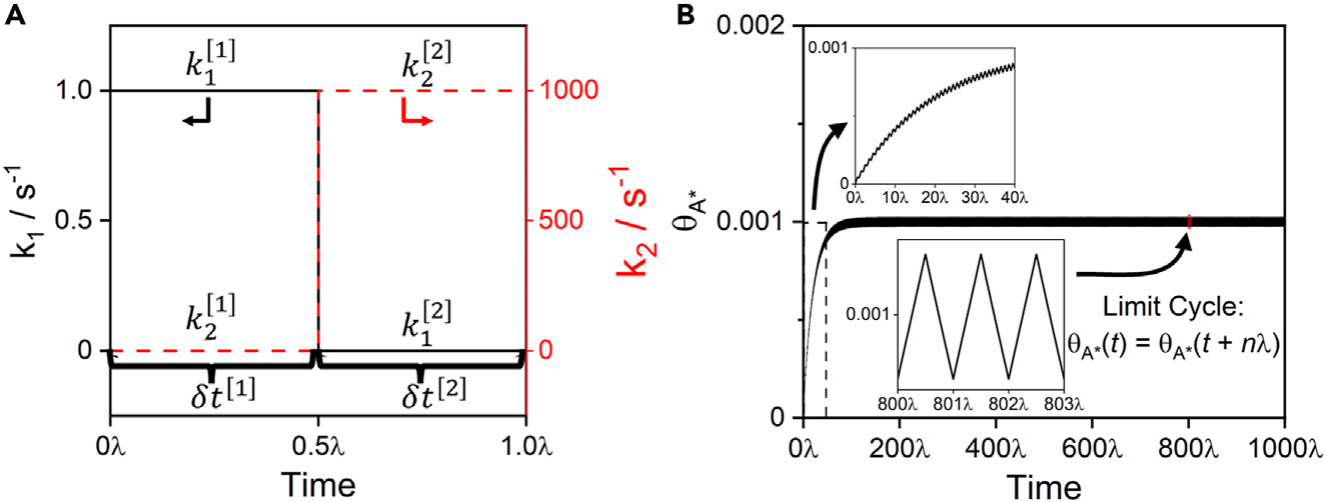
Example programmed catalysis oscillation (A) Oscillation in the magnitude of elementary step rate constants, $k_{1}$ and $k_{2}$, between state [1] and state [2]. (B) Convergence to the limit cycle from a clean surface ($\theta_{*}=1$) during programmed catalysis for a symmetric oscillation between the kinetic states in Scheme 1 with $\lambda =10^{-4}$ s and $a_{A}=1$.

We establish the conceptual and mathematical underpinnings of analyses hereinafter through examination of the simplest catalytic reaction suitable for rate enhancement via programmed catalysis—the two-step irreversible sequence A + ∗ → I^∗^ → B + ∗ (Scheme 1). We consider the case in which the catalytic reaction is purposefully oscillated between two states, [j], where either only step 1 (A + ∗ → I^∗^) or only step 2 (I^∗^ → B + ∗) is operative. During this catalytic program, elementary step rate constants $k_{i}^{\left[j\right]}$ for step i and state [j] oscillate with a wavelength λ (or frequency f=1/λ). In this example, the rate constants are $k_{i}=k_{i}^{\left[1\right]}$ for time $\delta t^{\left[1\right]}=\lambda /2$ followed by $k_{i}=k_{i}^{\left[2\right]}$ for time $\delta t^{\left[2\right]}=\lambda /2$, as illustrated in Figure 1A.

Without oscillation, the static steady-state rate, $r_{\text{ss}}$, for the reaction network in Scheme 1 in terms of the thermodynamic activity of A, $a_{A}$, is $r_{\text{ss}}=k_{1}k_{2}a_{A}/\left(k_{1}a_{A}+k_{2}\right)$, which is zero for both state [1] and [2] since one of the steps is “turned off” in either state. However, by oscillating between the two states, a catalytic program can couple step 1 in state [1] with step 2 in state [2] to give a nonzero reaction rate. Nonzero reaction rates are achieved by programmatically operating at state [1] to accumulate I∗ on the surface and then switching to state [2] to convert I∗ to B. Figure 1B illustrates the oscillatory response of I∗ coverage caused by the periodic switch between states [1] and [2] (Figure 1A).

The surface concentration history in Figure 1B is determined by forward integration of the differential equation describing the rates of $I^{*}$ generation and consumption (Equation 1):

(Equation 1) $$\frac{d\theta_{I^{*}}}{dt}=-k_{2}\left(t\right)\theta_{I^{*}}+k_{1}\left(t\right)a_{A}\theta_{*}=-k_{2}\left(t\right)\theta_{I^{*}}+k_{1}\left(t\right)a_{A}\left(1-\theta_{I^{*}}\right)$$

where $\theta_{*}+\theta_{I^{*}}=1$, $k_{i}=k_{i}^{\left[1\right]}$ for nλ≤t<(n+1/2)λ and $k_{i}=k_{i}^{\left[2\right]}$ for (n+1/2)λ≤t<(n+1)λ, with the initial condition $\theta_{I^{*}}\left(t=0\right)=0$. After hundreds of wavelengths, the fractional coverage of I∗ converges to a periodic limit cycle where (Equation 2):

(Equation 2) $$\theta_{I^{*}}\left(t\right)=\theta_{I^{*}}\left(t+n\lambda \right)$$

Forward integration (e.g., of Equation 1) is quantitatively accurate, but does not provide the same physical insight as an analytical solution and needlessly computes the entire transient when only the limit cycle is of interest.

To overcome these challenges, we develop a method for finding analytical solutions for the limit cycle directly by: (i) solving Equation 1 in piecewise fashion on the ranges from 0 to $\delta t^{\left[1\right]}$ and from $\delta t^{\left[1\right]}$ to $\delta t^{\left[1\right]}+\delta t^{\left[2\right]}$, and (ii) imposing continuity and periodic boundary conditions that define the limit cycle (Equation 2). The time-averaged rate during the limit cycle is given as Equation 3 (see the detailed derivation in Section S2 of the supplemental information):

(Equation 3) $$\left\langle r\right\rangle =\frac{\left(1-\exp\left(-k_{1}^{\left[1\right]}a_{A}{\delta t}^{\left[1\right]}\right)\right)\left(1-\exp\left(-k_{2}^{\left[2\right]}{\delta t}^{\left[2\right]}\right)\right)}{\left({\delta t}^{\left[1\right]}+{\delta t}^{\left[2\right]}\right)\left(1-\exp\left(-k_{1}^{\left[1\right]}a_{A}{\delta t}^{\left[1\right]}-k_{2}^{\left[2\right]}{\delta t}^{\left[2\right]}\right)\right)}$$

**Figure 2:**
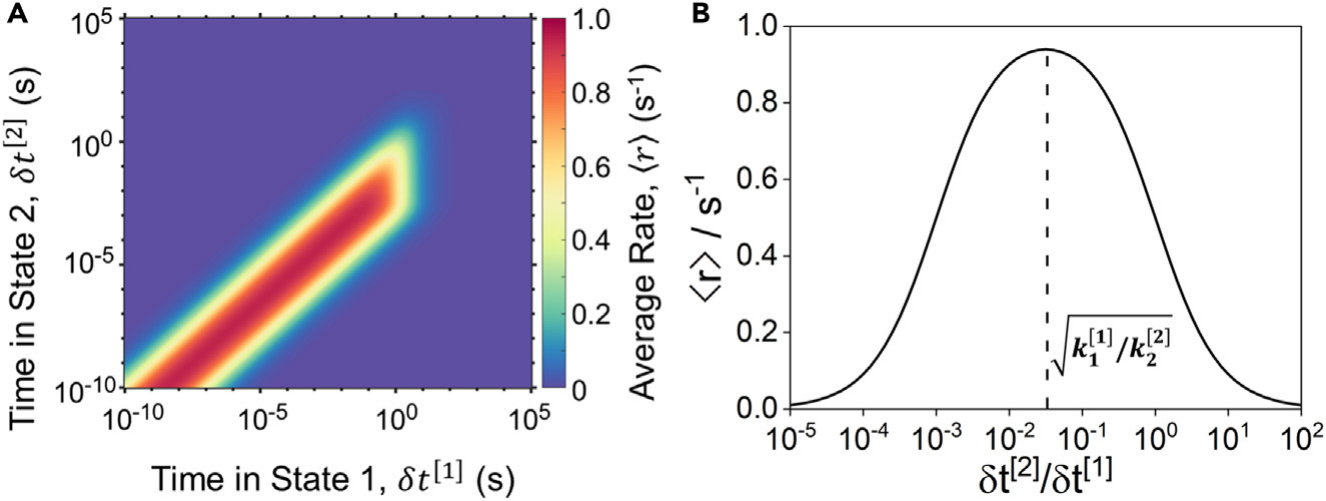
Programmed catalysis rates (A) Contour plot of the time-averaged rate as a function of time spent in state [1] $\left(\delta t^{\left[1\right]}\right)$ and [2] $\left(\delta t^{\left[2\right]}\right)$ for the reaction in Scheme 1 with $a_{A}=1$. (B) Time-averaged rate as a function of the ratio of time in state [2] to state [1] $\left(\delta t^{\left[2\right]}/\delta t^{\left[1\right]}\right)$ during quasi-static surface conditions.

The functional form of Equation 3 demonstrates that this system does not exhibit a finite optimal frequency band, as illustrated in Figure 2A. The only condition relevant to rate enhancement for this system is whether the oscillation is sufficiently fast, such that $k_{1}^{\left[1\right]}a_{A}{\delta t}^{\left[1\right]}\ll 1$ and $k_{2}^{\left[2\right]}{\delta t}^{\left[2\right]}\ll 1$. At these conditions, the surface coverage is approximately constant because the oscillation frequency is much faster than the time required for the surface coverage to change. We term this condition the “quasi-static surface condition” or “quasi-stasis”, for which Equation 3 simplifies to Equation 4, which is a function only of the ratio ${\delta t}^{\left[2\right]}/{\delta t}^{\left[1\right]}$, with time-averaged rates shown in Figure 2B.

(Equation 4) $$\left\langle r\right\rangle \approx \frac{k_{1}^{\left[1\right]}a_{A}k_{2}^{\left[2\right]}\left(\frac{{\delta t}^{\left[2\right]}}{{\delta t}^{\left[1\right]}}\right)}{\left(1+\frac{{\delta t}^{\left[2\right]}}{{\delta t}^{\left[1\right]}}\right)\left(k_{1}^{\left[1\right]}a_{A}+k_{2}^{\left[2\right]}\left(\frac{{\delta t}^{\left[2\right]}}{{\delta t}^{\left[1\right]}}\right)\right)}$$

Examination of Equation 4 reveals that, in general, the optimal ratio of ${\delta t}^{\left[2\right]}/{\delta t}^{\left[1\right]}$ is $\sqrt{k_{1}^{\left[1\right]}/k_{2}^{\left[2\right]}}$, as is evidenced by maximum rate occurring for $\delta t^{\left[2\right]}/\delta t^{\left[1\right]}=10^{-1.5}$ (Figure 2B). Identification of this simple, consequential mathematical relationship is made possible by the analytical solution and demonstrates that (i) synergistic asymmetry in rate constants and forcing program is key in determining the optimality of rate enhancement and (ii) the phenomenon of optimal frequency bands is not a general, or defining, feature of programmable catalysis. In the following, we generalize the presented analytical technique by development of an algorithmic procedure for reactions of any number of steps, network connectivity, and forcing program to calculate the time-averaged rates during programmed catalysis at minimal computational cost.

### Rapid calculation of limit cycles during programmable catalysis

The procedure employed for finding the limit cycle is generalizable to more complex reaction networks for any waveform of forced oscillation. In reaction schemes that do not involve the reaction between two species changing in time (e.g., bimolecular surface reaction steps), the coupled differential equations that describe the dynamics of fractional coverages are written in matrix form as (Equation 5):

(Equation 5) $$\frac{d}{dt}\theta^{\prime }=A^{\prime }\left(t\right)\theta^{\prime }+b^{\prime }\left(t\right)$$

where $\theta^{\prime }$ is a vector of all surface species (excluding vacant sites) and the matrix $A^{\prime }$ and vector $b^{\prime }$ are time-dependent functions of all relevant rate constants and chemical activities of fluid-phase species. The time-dependency of $A^{\prime }$ and $b^{\prime }$ is removed by separating Equation 5 into *n*-piecewise constant coefficient ODEs, one for each kinetic state of the catalytic program. The limit cycle is then readily found by linear algebra methods after application of periodic and continuity boundary conditions to find the constants of integration to obtain a piece-wise solution of the form (Equation 6) (for full derivation, see Section S3 of the supplemental information):

(Equation 6) $$\theta \left(t\right)=V^{\left[j\right]}\;\exp\left(\Lambda^{\left[j\right]}t\right)c^{\left[j\right]}+p^{\left[j\right]}\;\text{for}\;t\;\epsilon \;t^{\left[j\right]}$$

where $V^{\left[j\right]}$ and $\Lambda^{\left[j\right]}$ are the eigenvector and diagonal eigenvalue matrix of ${A^{\prime }}^{\left[j\right]}$, $c^{\left[j\right]}$ is the vector of integration constants, and $p^{\left[j\right]}$ is the particular solution of the ODE. The superscript indices all indicate these variables are in reference to the form of Equation 5 during the time in kinetic state [j]. The catalytic program can have any number of states, such that continuous oscillations (e.g., sine waves) are well approximated by many-state square waves. The solution gives $\theta^{\prime }\left(t\right)$ at the limit cycle, and the time-averaged rate is given by (Equation 7).

(Equation 7) $$\left\langle r\right\rangle =\frac{\int_{0}^{\lambda }r\left(\theta^{\prime }\left(t\right)\right)\;dt}{\lambda }$$

For non-linear reaction schemes, this approach is supplemented by Carleman linearization,^38^ which transforms non-linear differential equations into the form of Equation 5 by treating higher order terms (e.g., ${\theta_{I^{*}}}^{2}$) as variables whose time derivatives are functions of known lower order time derivatives (e.g., $\frac{d}{dt}{\theta_{I^{*}}}^{2}=2\theta_{I^{*}}\frac{d}{dt}\theta_{I^{*}}$).

Some non-linear reaction systems are not amenable to linearization techniques or require intractably high order approximations. This can occur for systems in which multiple limit cycles exist.^39^ In such instances, the limit cycle is instead found by formulating the periodic boundary condition as an optimization problem with the objective function in Equation 8:

(Equation 8) $$\min_{\theta \left(t=0\right)}\left\|\theta \left(t=0\right)-\int_{0}^{\lambda }\frac{d\theta }{dt}dt\right\|$$

where θ is a vector of all surface species and t=0 is the time at the start of the limit cycle. The integral calculates θ(t=λ), and the objective function minimizes the magnitude of the vector resulting from the difference θ(t=0)−θ(t=λ). This minimization problem is readily solved by gradient descent, Newton-Raphson, or forward integration approaches, which we demonstrate for a reaction with multiple limit cycle solutions in Section S4 of the supplemental information and apply to CO oxidation in the “programming enhanced rates of catalytic CO oxidation on Pt (111) surfaces” section.

In what follows, we apply the aforementioned techniques to rapidly calculate limit cycles of catalytic programs for linear/unimolecular and non-linear/bimolecular reaction schemes. These case studies not only demonstrate the computational efficiency of the techniques in Equations 5, 6, 7, and 8, but also serve to clarify the mechanism of rate enhancement via stepwise programmed catalysis. In the “mechanistic underpinnings of rate enhancement during programmable catalysis” section, we synthesize the learnings from these two case studies to establish general design principles for rationally promoting catalysis via either stepwise or quasi-static mechanisms.

### Illustrative case studies of programmable catalysis

**Scheme 2:**
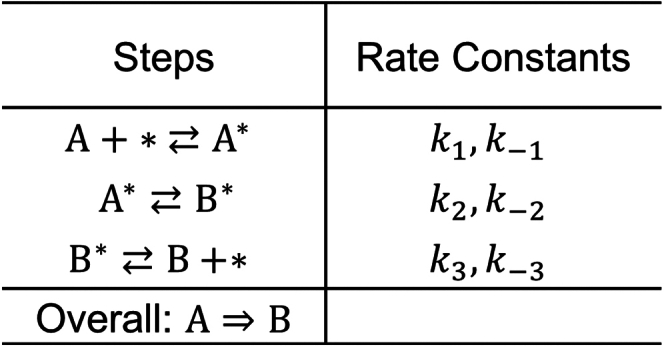
Linear three-step reaction network

We demonstrate the ease-of-use and mechanistic interpretability of the matrix-based approach embodied by Equations 5, 6, 7, and 8 through examination of the unimolecular three-step sequence A⇒B summarized in Scheme 2. This A⇒B reaction is one of the simplest catalytic sequences that exhibit optimal programmed catalysis performance within a finite frequency band^16^ and is an instructive example for understanding more generally the phenomenon of programmed catalysis. The imposition of LFE relationships, where transition state and binding energies are linearly interrelated, confers a maximum rate on A⇒B catalysis under static conditions. This Sabatier maximum rate can be overcome by programmatically enforcing an oscillation of the reaction coordinate potential energy surface in accordance with LFE scaling parameters.^31^ As an example, the relationship between adsorbate binding energies may be given by (Equation 9):^12^^,^^29^^,^^31^

(Equation 9) $$BE_{A}=\frac{BE_{B}-\left(1-\gamma \right)\delta -\Delta H_{\text{ovr}}}{\gamma }$$

where $\Delta H_{\text{ovr}}$ is the reaction enthalpy change, $BE_{A}$ and $BE_{B}$ are, respectively, the adsorption enthalpies of species A and B, and γ and δ are scaling parameters that dictate the magnitude and polarity of the relationship between binding energies. For example, (i) if $BE_{A}=\delta$, then the surface reaction is isothermic $\left(H_{A^{*}}=H_{B^{*}}\right)$ and (ii) the change in binding energy of A and B are related by $\gamma \Delta BE_{A}=\Delta BE_{B}$. Finally, the activation energy of the surface reaction is (Equation 10):

(Equation 10) $$E_{a,\text{sr}}=\alpha \Delta H_{\text{sr}}+\beta$$

where $\Delta H_{\text{sr}}$ is the enthalpy change of the surface reaction (A∗ to B∗). In the simplest case considered here, adsorption of A and B are treated as barrierless reactions, which is reasonable for non-dissociative adsorption when the transition state closely resembles the gas-phase species. Typical pre-exponential factors of 10^6^ s^−1^ and 10^13^ s^−1^ are used for adsorption and desorption/surface reaction, respectively, with gas-phase activities referenced to 1 bar.

**Figure 3:**
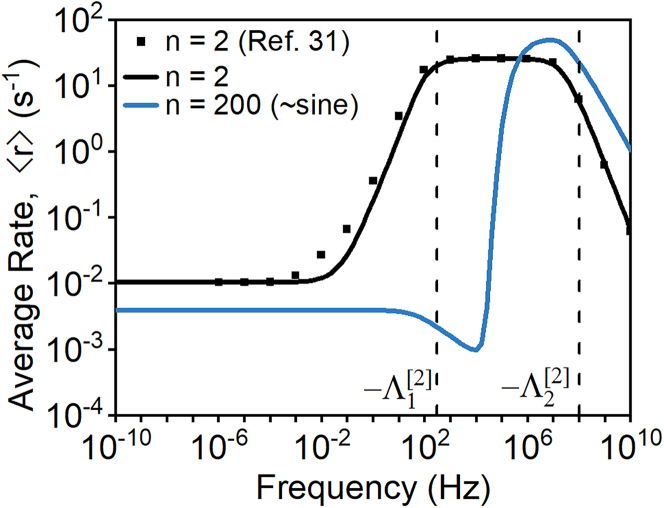
Time-averaged rate as a function of frequency for a 2-state square wave and a 200-state square wave that approximates a sine wave Black squares are the time-averaged rates reported by Ardagh et al.^31^ for a square wave with n = 2. For these calculations, γ=0.5, δ=1.4eV, $\Delta H_{\text{ovr}}=0\;\text{eV}$, α=0.6, β=102kJ/mol. The binding energy $BE_{B}$ is purposefully oscillated from 0.1 to 1.03 eV. See also Section S5 in the supplemental information.

We employ the matrix-based methodology described heretofore to calculate time-averaged rates and adsorbate surface coverages during limit cycles with oscillation frequencies between 10^−10^–10^10^ Hz for a 2-state square-wave and a sine wave approximated by a 200-state square wave. The case of n *=* 2 corresponds to the square waveform and is used here to validate against previously reported^31^ numerical forward integration results. As shown in Figure 3, our calculations (black line) agree excellently with these previous results^31^ (black squares) and, crucially, calculates ⟨r⟩ more than 8 orders of magnitude faster than forward integration strategies (see sections S4 and S5 of the supplemental information for details). Figure 3 also shows ⟨r⟩ calculated for sinusoidal oscillations approximated by 200-state square waves. Approximation of sine waves with n = 200 states is nearly identical to n = 10,000 states and is calculated with a median time of ∼30 ms irrespective of oscillation frequency.

The matrix-based approach produces semi-analytical piece-wise solutions for surface coverage in the form of Equation 6, where calculation of the eigenvalues, $\Lambda^{\left[j\right]}$, and eigenvectors, $V^{\left[j\right]}$, typically require numerical calculation. The relationship between eigenvectors/eigenvalues and surface coverage, however, is in exclusive terms of elementary functions and, therefore, readily interpretable. Indeed, the functional form of Equation 6 makes clear that eigenvalues, $\Lambda^{\left[j\right]}$, quantify the characteristic timescales of programmed catalysis and are consequently related to the band of optimal frequencies in Figure 3.

**Figure 4:**
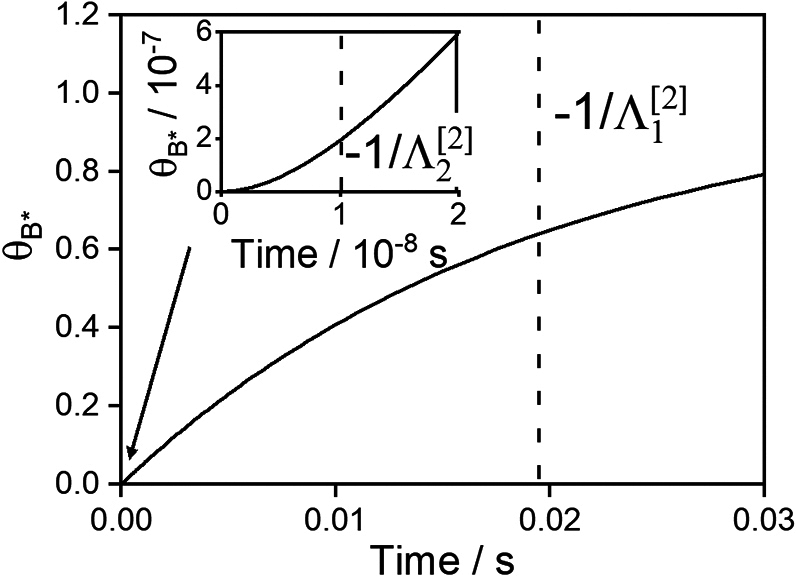
The fractional coverage of B∗ as a function of time during kinetic state [2] of the limit ${\text{BE}}_{R}^{\left[1\right]}=0.1\;\text{eV}$, ${\text{BE}}_{R}^{\left[2\right]}=1.03\;\text{eV}$, $P_{A}=100_{\text{bar}}$, $P_{B}=0$. The inverse eigenvalues, which define characteristic frequencies, are shown by vertical dashed lines. Inset: A closer view from 0 to 2 × 10^−8^ s.

In particular, from examination of the temporal evolution of B∗ coverage (Figure 4), we infer that optimal frequency bands arise from a balance between two characteristic timescales in state [2] of the catalytic program, during which the binding energy of B∗ is large and, accordingly, B∗ is accumulated on the surface. During this period, the catalyst first (i) binds A to the surface as A∗ and then (ii) converts A∗ into B∗. Thus, sufficient time in state [2] must be allowed for these two kinetic processes to occur, but the duration of state [2] must be small enough that steady-state catalysis is not reached. The temporal efficiency of the net conversion of A to B∗ during state [2] is therefore optimized when $\delta t^{\left[2\right]}$ lies between (i) the initiation of rapid B∗ generation and (ii) the onset of steady-state catalysis. As shown in Figure 4, these two timescales, respectively, correspond to $t=-1/\Lambda_{2}^{\left[2\right]}$ and $t=-1/\Lambda_{1}^{\left[2\right]}$—the inverse of the two eigenvalues of state [2] determined from the governing ODE matrix (i.e., $A^{\prime \left[2\right]}$ in Equation 5). As shown in Figure 3, these eigenvalues bound the optimal frequency band, corroborating identification of $-1/\Lambda_{2}^{\left[2\right]}$ and $-1/\Lambda_{1}^{\left[2\right]}$ as the relevant timescales for the catalytic program.

In the following, we demonstrate how the mechanistic understanding of stepwise programmed catalysis developed in the foregoing analyses can be readily applied to more complex, industrially relevant reactions such as CO oxidation on Pt (111) surfaces. Despite the increased complexity arising from non-linearity in the form of bimolecular steps in CO oxidation (e.g., O∗ + CO∗ → CO_2_∗ + ∗), the mathematical methods established herein enable facile calculation of time-averaged rates across a wide parameter space.

#### Programming enhanced rates of catalytic CO oxidation on Pt (111) surfaces

Rates and reaction orders of catalytic CO oxidation by O_2_ on Pt surfaces are well-documented to be highly sensitive to the extent of surface strain.^40^^,^^41^^,^^42^^,^^43^^,^^44^ Grabow and co-workers^40^ have shown that compression and expansion of Pt (111) surfaces profoundly perturbs adsorbate binding energies and, consequently, results in two distinct kinetic regimes defined by rate-determining O_2_∗ dissociation (compressed surfaces) or CO∗ + O∗ combination (stretched surfaces). Therefore, rates of CO oxidation are optimized at an intermediate extent of lattice strain corresponding to the transition between rate-controlling steps per the Sabatier principle. From this well-established kinetic behavior, we infer that CO oxidation is ideally suited to rate enhancement through a catalytic program that purposefully oscillates between states which sequentially promote either O∗ formation or CO∗ + O∗ combination.

**Scheme 3:**
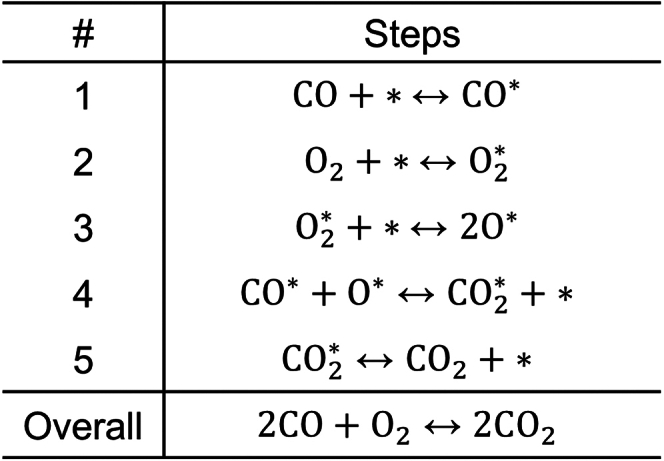
Catalytic CO oxidation on Pt (111) surfaces The dependencies of each elementary step rate and/or equilibrium constant on lattice strain are detailed in Section S6.

We demonstrate the suitability of CO oxidation to stepwise programmed rate enhancement by leveraging the presented optimization method to calculate time-averaged rates and characteristic timescales of the non-linear elementary step reaction sequence summarized in Scheme 3. Kinetic parameters as a function of lattice strain are determined from DFT-calculated free energies reported by Grabow et al.^40^

**Figure 5:**
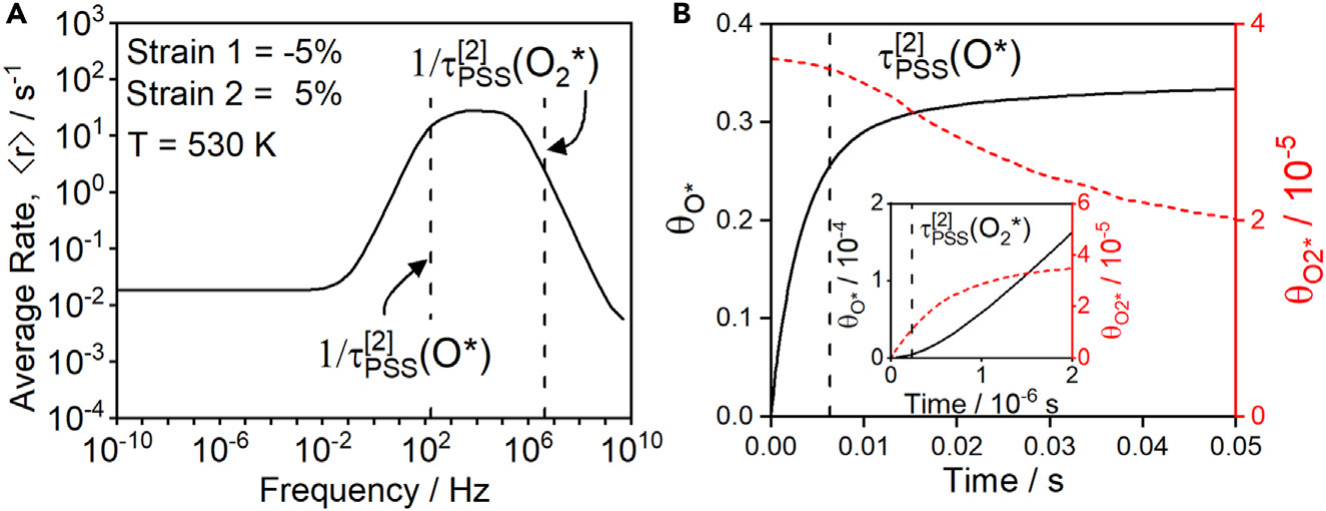
Programmed catalysis for CO oxidation on Pt(111) (A) Average rate versus frequency for CO oxidation on Pt for a symmetric oscillation in strain from −5 to +5% at 530 K. Vertical dashed lines indicate the timescales for pseudo-steady-state (PSS) to describe O∗ and O_2_∗ surface coverages in state [2]. (B) Time evolution of O∗ and O_2_∗ during the limit cycle at 5 Hz in state [2]. The surface species CO∗ and ∗ are quasi-static with fractional coverages of ∼0.63 and 0.36, respectively. The PSS timescales for O∗ and O_2_∗ are approximately $k_{1}^{\left[2\right]}a_{O_{2}}$ and $k_{3}^{\left[2\right]}$, respectively.

Figure 5 shows rates of programmed CO oxidation catalysis with lattice strain purposefully varied between −5% and +5% at 530 K and with 1.02 Pa O_2_ and 0.19 Pa CO, in accordance with experimental conditions of Cirak et al.^41^ Forcing programs applied in Figure 5 are symmetric, with frequencies spanning from 10^−10^ Hz–10^10^ Hz. Akin to the preceding example of A⇒B catalysis, the rate of CO oxidation is optimally enhanced within a band of optimal frequencies, ∼10^2^–10^6^ Hz, that accelerate reaction ∼20× relative to the static Sabatier maximum (Figure 5A). The optimal frequency band for stepwise programmed catalysis is, again, quantitatively described by the characteristic timescales of two distinct elementary steps that must be executed within a single state of the forcing program. In particular, state [2] of the catalytic program is burdened with executing both the adsorption of O_2_ and the subsequent dissociation to form O∗. Correspondingly, the band of optimal frequencies in Figure 5A is bound by the characteristic timescales for surface coverages of O_2_∗ and O∗ to achieve pseudo-steady state (PSS). This interpretation of the optimal frequencies is corroborated by examination of the temporal evolution of O_2_∗ and O∗ surface coverages in Figure 5B. As shown in Figure 5B, O_2_∗ and O∗, respectively, reach pseudo-steady-state coverages at ∼10^−6^ s and ∼10^−2^ s. Programmed switching from +5% strain (state [2]) to −5% strain (state [1]) at frequencies between these two timescales efficaciously induces rapid catalysis of the CO∗ + O∗ reaction to form CO_2_∗, which readily desorbs to form CO_2_ to complete the catalytic cycle. Surface O∗ is then replenished by the rate-controlling steps present in state [2] as the cycle repeats.

The exhaustive calculation of programmed rates of CO oxidation catalysis as a function of applied lattice strain in Figure 5A is made possible by the computational efficiency of the optimization method described previously. Indeed, the mathematical tools described in this work theoretically enable facile exploration of the entire parameter space available for optimization of programmed CO oxidation—which includes, for example, shape of forcing waveform (e.g., sine wave), temporal asymmetry of forcing waveform, reaction temperature, and reactant partial pressures. To most clearly demonstrate the utility of the developments herein for rapid optimization of programmed catalysis, we return to the precedent example of the A⇒B reaction (Scheme 2).

#### Calculating the optimal catalytic program to maximize reaction rate

Maximizing the time-averaged rate of the A⇒B reaction requires optimization of the four parameters that define the programmed waveform—the B∗ binding energy and the duration spent in each of the two states of the program. By combining the matrix-based analyses with gradient-descent methods, we solve this four-parameter optimization problem in 100 ms and discover the *global* maximum rate of 382 s^−1^ with ${\text{BE}}_{B}^{\left[1\right]}=0.1\;\text{eV}$ for $\delta t^{\left[1\right]}=10^{-4}\lambda$ and ${\text{BE}}_{B}^{\left[2\right]}$ = 0.9 eV for $\delta t^{\left[2\right]}=0.9999\lambda$, where f=1/λ=6.1MHz. We verify that this catalytic program is likely the global maximum by comparison to an unrestrained 20-step waveform, which converges to the same solution. Importantly, this optimal waveform spends 10^4^× longer in state [2] compared to state [1], which is consistent with the rate-controlling processes occurring exclusively in state [2]. Moreover, the time-averaged rate in the optimal program is ∼14× larger than the maximum for a symmetric square wave with ${\text{BE}}_{B}^{\left[1\right]}=0.1\;\text{eV}$ and ${\text{BE}}_{B}^{\left[2\right]}=1.03\;\text{eV}$ (Figure 3)—emphasizing the significant improvements achieved through combination of mathematical and conceptual developments.

**Figure 6:**
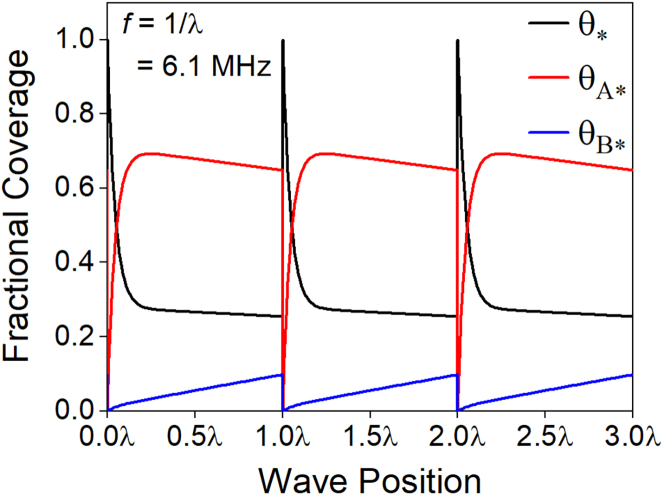
Fractional coverages as a function of wave position for the optimal program with ${\text{BE}}_{B}^{\left[1\right]}=0.1\;\text{eV}$ for $10^{-4}\lambda$ and ${\text{BE}}_{B}^{\left[2\right]}$ = 0.9 eV for 0.9999 λ, where f=1/λ=6.1MHz The time-averaged rate at these conditions is ∼382 s^−1^.

The molecular-level dynamics that underpin the optimal catalytic program are similar to those embodied in Figures 3 and 4, except for the asymmetry in the duration of each step of the square waveform. The optimized waveform leverages the rapidity of B∗ to B desorption in the low binding energy state, state [1], by programming state [1] to be 10^4^× shorter than the high binding energy state, state [2]. As shown in Figure 6, the optimal waveform rapidly generates a bare catalyst surface in kinetic state [1] while state [2] slowly accumulates B∗ until a coverage of $\theta_{B^{*}}∼\;0.1$, then repeats indefinitely. This temporal asymmetry of the waveform increases the time-averaged rate by ∼2× by minimizing the temporal losses of operating at kinetic state [1] longer than necessary. The remaining ∼7× increase is achieved through optimal choice of ${\text{BE}}_{B}^{\left[1\right]}$ and ${\text{BE}}_{B}^{\left[2\right]}$.

From the analyses heretofore, we put forth a general conceptual grounding of stepwise programmed catalysis in the following. Simply put, a catalytic program is a sequence of instructions, each defining two parameters: a potential energy surface (e.g., $BE_{B}^{\left[j\right]}$) and a hold time (e.g., $\delta t^{\left[j\right]}$). Correspondingly, each instruction in the kinetic program executes a segment of the catalytic cycle, and each instruction of the program must be tailored to its task. Under this view, the optimal waveform is easily rationalized. For the example in Figure 6, state [1] is executed to convert B∗ to ∗ and therefore the optimal potential energy surface (PES) has a low binding energy at the prescribed lower bound of 0.1 eV to maximize the rate constant for B∗ desorption ($k_{3}^{\left[1\right]}$). In turn, the optimal hold time in this state, $\delta t^{\left[1\right]}=1.6\times 10^{-11}\;s\text{,}$ is commensurate with $1/k_{3}^{\left[1\right]}$, an eigenvalue of state [1] describing the timescale for B∗ desorption. Similarly, state [2] is executed to convert A + ∗ to B∗ and, therefore, the optimal time $\delta t^{\left[2\right]}$ is longer than the timescale of pseudo-steady-state for A∗ but shorter than the timescale for reaching steady-state catalysis. The optimality of balancing $\delta t^{\left[2\right]}$ between these two characteristic timescales is in direct accordance with the task assigned to state [2]. The PES in state [2] must also balance the rate of the $A^{*}\to B^{*}$ surface reaction against the quasi-equilibrated surface coverage of A∗; therefore, state [2] is optimized with an intermediate binding energy, ${\text{BE}}_{B}^{\left[2\right]}=0.9\;\text{eV}$.

From this understanding, we suggest that considering waveforms in terms of their frequency and amplitude obfuscates the salient parameters of the kinetic programs, which are the individual timescales and potential energy surfaces for each kinetic state, or instruction in the program. We refer to these mechanisms as stepwise programmed catalysis to emphasize that each instruction in the kinetic program is tailored to its segment of the catalytic cycle. This view of kinetic programs also suggests that optimal waveforms are likely those consisting of a small number of kinetic states in the form of a square wave, and we conjecture that the number of unique kinetic states in the optimal waveform is at most equal to the number of steps in the reaction network.

The optimality of intermediate ${\text{BE}}_{B}^{\left[2\right]}$ suggests that, despite accelerating rate beyond static maxima, stepwise programs remain subject to Sabatier limits. The manifestation of this principle is evinced by derivations of rate functions in the following sections. In foregoing analyses closed-from rate functions revealed that some catalytic systems are optimally stimulated by oscillations at the high frequency limit, where surface coverages become quasi-static. For these programs, the order of "instructions" is unimportant because the effective kinetics of the reaction system are a temporal average of each kinetic state. Motivated by these insights, we seek to establish a general understanding of the consequences of LFE scaling relationships on (i) mechanisms of programmable rate enhancement and (ii) limits to rate enhancement achievable through programmed catalysis. In so doing, we identify the distinct energetic requirements that, respectively, favor quasi-static or stepwise mechanisms in explicit terms of LFE scaling parameters for the reaction in Scheme 2. In addition to specifying these criteria, we derive analytical equations for time-averaged rate that are identical in functional form and physical interpretability to conventional rate equations for static catalysis.

### Mechanistic underpinnings of rate enhancement during programmable catalysis

To formulate general design principles that dictate the suitability of catalytic systems to rate enhancement via stepwise or quasi-static mechanisms, we reconsider the three-step reaction in Scheme 2. The analyses of Scheme 2 in the section “illustrative case studies of programmable catalysis” considered only a single set of LFE parameters that determined the kinetic behavior of the A⇒B reaction which, for the preceding examples, exhibited rate enhancement via stepwise programs. By investigating the entire parameter space of LFE relationships, we discover the disparate conditions which enable promotion by either mechanism. These learnings are enabled by general description of the interplay of LFE scaling parameters and free energy reaction barriers in terms of $\omega_{j}$, defined by (Equation 11):

(Equation 11) $$\omega_{j}=\frac{\Delta \;\ln\;k_{j}}{\Delta \;\ln\;k_{3}}\overset{\Delta T=0}{=}\frac{\Delta {\Delta G}_{j}^{o‡}}{{\Delta \text{BE}}_{B}}$$

where $\Delta {\Delta G}_{j}^{o‡}$ is the change in the activation free energy for step j ($\Delta {\Delta G}_{j}^{o‡}={\Delta G}_{j}^{o‡\left[2\right]}-{\Delta G}_{j}^{o‡\left[1\right]}$) and ${\Delta \text{BE}}_{B}$ is the oscillation amplitude for the change in the binding energy of the product, B. We note that this formulation is equivalent to the α-γ formalism used in Equations 9 and 10 with $\omega_{-1}=1/\gamma$ and $\omega_{2}=\alpha \left(1/\gamma -1\right)$, where $\gamma \equiv \Delta BE_{B}/\Delta BE_{A}$, which can take any value, and $\alpha \equiv \Delta E_{a,\text{sr}}/\Delta H_{\text{sr}}$, which precedent literature^45^ suggests is typically bounded from 0 to 1, though exceptions likely exist. For the sake of generality, we allow $\omega_{j}$ to vary beyond the bound 0<α<1. Recalling that adsorption of A and B are barrierless, $k_{1}=k_{-3}$ are not functions of ${\Delta \text{BE}}_{B}$ and thus $\omega_{1}=\omega_{-3}=0$. Finally, because the overall reaction free energy change is constant during variation of $BE_{B}$, the values of $\omega_{j}$ are constrained by the equality $\sum_{j<0}\omega_{j}=\sum_{j>0}\omega_{j}$. Thus, we define $\omega_{-2}=\omega_{3}+\omega_{2}-\omega_{-1}$ to ensure thermodynamic consistency, where $\omega_{3}=1$ by the definition in Equation 11.

With these constraints, there are two independent scaling parameters: $\omega_{-1}$ and $\omega_{2}$. The former, $\omega_{-1}=\Delta BE_{A}/\Delta BE_{B}$, reflects the magnitude and polarity of correlation between A∗ and B∗ binding energies. The latter, $\omega_{2}=\Delta {\Delta G}_{2}^{o‡}/\Delta BE_{B}$, reflects the degree of correlation between the kinetic barrier of step 2 ($A^{*}\to B^{*}$) and the binding energy of B∗. The interplay of these two scaling parameters and the program waveform, $BE_{B}\left(t\right)$, fully describe the sensitivity of the A⇒B reaction to $BE_{B}$ oscillation and predict the optimal mechanisms of rate enhancement. We systematically catalog the values of $\omega_{-1}$ and $\omega_{2}$ that enable acceleration of rate by re-examining our physical, mechanistic interpretations of quasi-static and stepwise programs for rate enhancement in the following sections.

#### Linear free energy relationships for quasi-static programmable catalysis mechanisms

The quasi-static condition is met when oscillation frequencies are sufficiently fast that the surface coverages of all intermediates are static in time. Under these conditions, the molecular-level dynamics of catalysis are describable in terms of time-averaged rate constants, $\left\langle k_{j}\right\rangle$. Consequently, the effective “equilibrium constant” for programmed catalysis under quasi-static conditions, $K_{P}$, can be quantified as (Equation 12).

(Equation 12) $$K_{P}=\frac{\left\langle k_{1}\right\rangle \left\langle k_{2}\right\rangle \left\langle k_{3}\right\rangle }{\left\langle k_{-1}\right\rangle \left\langle k_{-2}\right\rangle \left\langle k_{-3}\right\rangle }$$

Equation 12 is identical to formulation of the conventional thermodynamic equilibrium constant, K, except that $K_{p}$ is a product of *time-averaged* rate constants $\left\langle k_{j}\right\rangle$. Crucially, Equation 12 implies that if $K_{p}>K$, then a positive time-averaged reaction rate is achievable even when reactants and products are present in their thermodynamic equilibrium ratios. The consequences of this condition in explicit terms of LFE scaling relations are evident upon expansion of $\left\langle k_{j}\right\rangle$ in terms of $\omega_{j}$ and the oscillation amplitude ${\Delta \text{BE}}_{B}$ to produce an equation for $K_{p}$ in terms of K (Equation 13).

(Equation 13) $$\frac{K_{p}}{K}=\frac{\left(1+\frac{\delta t^{\left[2\right]}}{\delta t^{\left[1\right]}}\exp\left(-\omega_{2}\frac{\Delta {\text{BE}}_{B}}{RT}\right)\right)\left(1+\frac{\delta t^{\left[2\right]}}{\delta t^{\left[1\right]}}\exp\left(-\frac{\Delta {\text{BE}}_{B}}{RT}\right)\right)}{\left(1+\frac{\delta t^{\left[2\right]}}{\delta t^{\left[1\right]}}\exp\left(-\omega_{-1}\frac{\Delta {\text{BE}}_{B}}{RT}\right)\right)\left(1+\frac{\delta t^{\left[2\right]}}{\delta t^{\left[1\right]}}\exp\left(-\left(1+\omega_{2}-\omega_{-1}\right)\frac{\Delta {\text{BE}}_{B}}{RT}\right)\right)}$$

Equation 13 reveals that, for appropriate $\omega_{-1}$ and $\omega_{2}$, programmable catalysis can drive the A⇒B reaction against thermodynamic gradients by input of thermodynamic work through variation of adsorbate free energies. Indeed, Equation 13 closely resembles formulae derived by Astumian and coworkers^16^ that similarly describe enzymatic catalysis driving reactions against thermodynamic gradients. The remarkable feature of Equation 13 is that it not only identifies conditions suitable for supra-equilibrium catalysis, but also predicts which values of $\omega_{-1}$ and $\omega_{2}$ allow for rate enhancement via quasi-static mechanisms.

**Figure 7:**
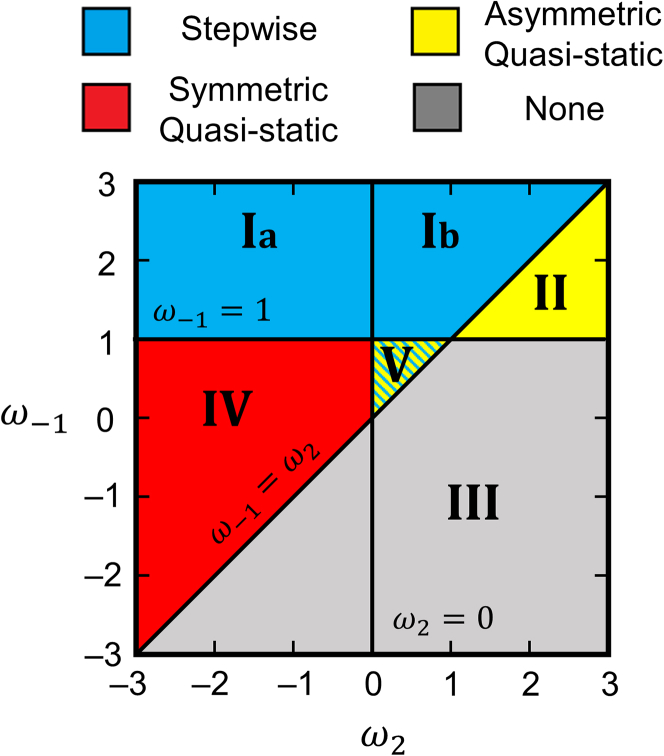
Categorization of mechanisms of optimal programmable rate enhancement via stepwise and/or quasi-static mechanisms Enhanced rates and supra-equilibrium conversion are achieved in Region I (blue) in an optimal frequency band by stepwise mechanisms, Region II (yellow) with an asymmetric square wave at quasi-static surface frequencies, and Region IV (red) with symmetric square wave oscillation at quasi-static surface frequencies. In Region III (gray), programmable rate enhancement is not achievable through quasi-static or stepwise mechanisms. In Region V (blue and yellow), rate enhancement is achievable through both stepwise and asymmetric quasi-static mechanisms.

In particular, inspection of Equation 13 reveals the existence of two “iso-equilibrium” conditions for which $K_{p}=K$. Equivalence of programmed, $K_{p}$, and thermodynamic, K, equilibrium constants is achieved if either $\omega_{-1}=1$ or $\omega_{-1}=\omega_{2}$. Along each of these iso-equilibrium lines, shown in Figure 7, catalytic programs neither promote nor demote the effective driving force for catalysis. However, in the regions between these two bounds (Regions II, IV, and V in Figure 7), LFE scaling parameters encode for reaction schemes that are energetically well-suited for enhancement of reaction rate through quasi-static mechanisms.^46^ Specifically, evaluation of Equation 13 within Regions II, IV, and V produces $K_{p}\gg K$, indicating that high frequency oscillation of $BE_{B}$ reduces kinetic and thermodynamic barriers to catalysis by orders of magnitude.

These three regions are distinguished by the intersection of the iso-equilibrium lines and the condition $\omega_{2}=0$, either side of which the kinetic barrier for step 2 ($A^{*}\to B^{*}$) and the binding energy of B∗ ($BE_{B}$) are either negatively ($\omega_{2}<0$) or positively ($\omega_{2}>0$) correlated. The polarity of this scaling relationship ultimately dictates whether each region requires temporal asymmetry of $BE_{B}$ oscillations. Following the prescriptions of Equation 13, Regions II, V ($\omega_{2}>0$), and IV ($\omega_{2}<0$) are, respectively, promoted using asymmetric ($\delta t^{\left[1\right]}\neq \delta t^{\left[2\right]}$) and symmetric ($\delta t^{\left[1\right]}=\delta t^{\left[2\right]}$) forcing programs.

Within each of these regions, reaction rate is described using closed-form expressions identical to those for conventional steady-state catalysis—except that each rate constant is time-averaged. In particular, for the three-step reaction in Scheme 2, the general form of the rate function is given by Equation 14, where $M_{ij}$ is the value of matrix M at the index i,j.^47^

(Equation 14) $$\begin{array}{c} \left\langle r\right\rangle =\frac{\left\langle k_{1}\right\rangle \left\langle k_{2}\right\rangle \left\langle k_{3}\right\rangle a_{A}-\left\langle k_{-1}\right\rangle \left\langle k_{-2}\right\rangle \left\langle k_{-3}\right\rangle a_{B}}{\sum_{j}\sum_{i}M_{ij}}\\ M=\left[\begin{array}{ccc} \left\langle k_{2}\right\rangle \left\langle k_{3}\right\rangle & \left\langle k_{-1}\right\rangle \left\langle k_{3}\right\rangle & \left\langle k_{-1}\right\rangle \left\langle k_{-2}\right\rangle \\ \left\langle k_{3}\right\rangle \left\langle k_{1}\right\rangle a_{A} & \left\langle k_{-2}\right\rangle \left\langle k_{1}\right\rangle a_{A} & \left\langle k_{-2}\right\rangle \left\langle k_{-3}\right\rangle a_{B}\\ \left\langle k_{1}\right\rangle \left\langle k_{2}\right\rangle a_{A} & \left\langle k_{-3}\right\rangle \left\langle k_{2}\right\rangle a_{B} & \left\langle k_{-3}\right\rangle \left\langle k_{-1}\right\rangle a_{B} \end{array}\right] \end{array}$$

Identically to traditional Langmuir-Hinshelwood rate expressions, the denominator of Equation 14 is simplified by the existence of dominant terms corresponding to the rate-controlling steps and most abundant surface intermediates, reducing the equation to functional forms that are physically interpretable and reflect familiar kinetic behavior (e.g., changes in rate-determining step or saturation of surface intermediates).

**Figure 8:**
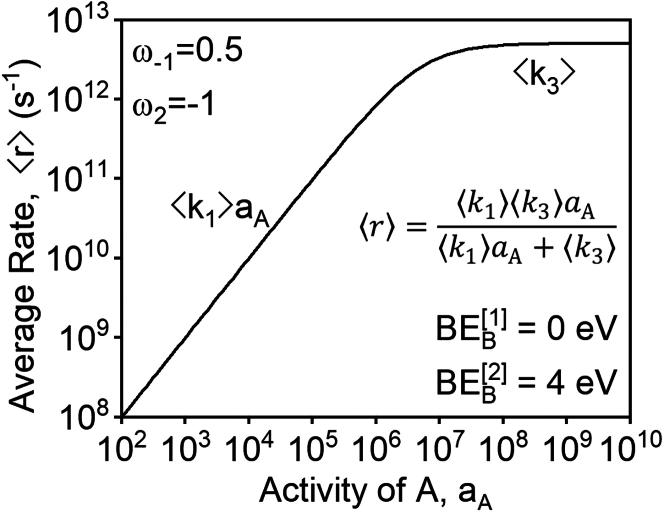
Quasi-static rate of catalysis for a catalyst in Region V ($\omega_{-1}=0.5,\omega_{2}=-1$) as a function activity of A for fixed binding energy of B in state [2]

We demonstrate the utility of Equation 14 by considering a simple forcing program for a catalytic system with LFE scaling parameters that lie in Region IV. Region IV defines the boundaries for which $K_{p}\gg K$, implying that the numerator in Equation 14 favors the forward reaction significantly more than steady-state catalysis. The denominator of Equation 14 is simplified by recognizing that for a symmetric, high-amplitude oscillation between ${\text{BE}}_{B}^{\left[1\right]}$ and ${\text{BE}}_{B}^{\left[2\right]}$, $\left\langle k_{j}\right\rangle \approx \frac{1}{2}k_{j}^{\left[1\right]}$ for $\omega_{j}>0$ and $\left\langle k_{j}\right\rangle \approx \frac{1}{2}k_{j}^{\left[2\right]}$ for $\omega_{j}<0$. Applying these constraints within Region IV necessitates that for sufficiently large ${\text{BE}}_{B}^{\left[2\right]}$ with fixed ${\text{BE}}_{B}^{\left[1\right]}$, the prevailing terms in M must be $\left\langle k_{1}\right\rangle \left\langle k_{2}\right\rangle a_{A}$ and $\left\langle k_{2}\right\rangle \left\langle k_{3}\right\rangle$, producing the closed-form rate expression shown in Figure 8.

Figure 8 demonstrates that, as predicted, the time-averaged rate of programmed A⇒B catalysis is well-described by a familiar analytical rate expression of the Langmuir-Hinshelwood form. Under quasi-static conditions engendered by high-frequency, high-amplitude oscillation, the identities of rate-controlling steps and most abundant surface intermediates are quantitatively predicted by the relative kinetic driving forces for step 1 and step 3. For $a_{A}≲10^{7}$, $\left\langle k_{3}\right\rangle \gg \left\langle k_{1}\right\rangle a_{A}$, resulting in rate-controlling A with a first-order dependence on the thermodynamic activity of A. For $a_{A}≳10^{7}$, the surface saturates in reactive intermediates and, correspondingly, the rate-determining step is $B^{*}$ desorption.

The predictive power of the closed-form rate law in Figure 8 exemplifies the utility of the framework embodied by Equation 13 and motivates exploration of regimes beyond those promotable by quasi-static catalysis. Indeed, by inspection of Equation 13 we see that, beyond Regions II, IV, and V of Figure 7, the quasi-static surface condition *demotes* catalysis, indicated by $K_{p}\ll K$. In these regimes and in the centralized Region V, we explore the capacity of stepwise programs to accelerate catalysis through further development of Langmuir-Hinshelwood-form expressions for reaction rate and surface coverage.

#### Linear free energy relationships for stepwise programmed catalysis

**Figure 9:**
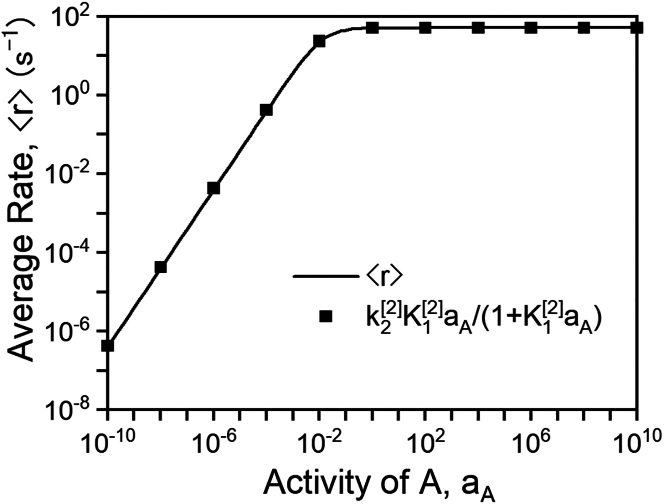
Comparison of numerical and closed-form reaction rates Numerically calculated time-averaged rate (solid line) compared to closed-form rate expression in Equation 16 (square points) for $BE_{B}^{\left[1\right]}=0\;\text{eV},BE_{B}^{\left[2\right]}=1.03\;\text{eV}$, δ=1.4eV, $\omega_{-1}=2$, $\omega_{2}=0.6$, $\delta t^{\left[2\right]}/\delta t^{\left[1\right]}=10^{6}$, $a_{B}=0$, $f=10^{3}\;\text{Hz}$.

As detailed hereinbefore, optimal frequency bands in stepwise programmed catalysis manifest if one state of a catalytic program is burdened with executing two elementary steps. In the case of the reaction in Scheme 2, the kinetic state with high binding energy of B is responsible for both adsorbing the reactant A (step 1) and converting A∗ to B∗ (step 2). We therefore surmise that the time-averaged rate of B formation, ⟨r⟩, must be equal to the time-averaged rate of B∗ formation, which occurs predominantly during state [2]. Thus, the time-averaged rate is approximated as (Equation 15):

(Equation 15) $$\left\langle r\right\rangle \approx \frac{\delta t^{\left[2\right]}}{\delta t^{\left[1\right]}+\delta t^{\left[2\right]}}r_{B^{*}}^{\left[2\right]}\approx \frac{k_{1}^{\left[2\right]}a_{A}k_{2}^{\left[2\right]}}{k_{1}^{\left[2\right]}a_{A}+k_{2}^{\left[2\right]}+k_{-1}^{\left[2\right]}+k_{-2}^{\left[2\right]}}$$

where $r_{B^{*}}^{\left[2\right]}$ is the rate of B∗ formation in state 2 and $\delta t^{\left[2\right]}\gg \delta t^{\left[1\right]}$ under optimal conditions. The familiar form of Equation 15 facilitates interpretation of stepwise programmed catalysis through well-established mechanistic understanding of rate expressions—identically to Equation 14. Depending on the prevailing terms in the denominator, Equation 15 will simplify to Langmuir-Hinshelwood-type expressions with varying rate-controlling steps, quasi-equilibrium assumptions, and most abundant surface intermediates. In the case where $k_{1}^{\left[2\right]}a_{A}+k_{-1}^{\left[2\right]}\gg k_{2}^{\left[2\right]}+k_{-2}^{\left[2\right]}$, the quasi-equilibrium assumption is valid on adsorption of A ($*+A↔A^{*}$) and the surface reaction ($A^{*}\to B^{*}$) is the rate-controlling step.^47^^,^^48^^,^^49^^,^^50^ The rate then becomes (Equation 16):

(Equation 16) $$\left\langle r\right\rangle \approx r_{B^{*}}^{\left[2\right]}\approx \frac{k_{2}^{\left[2\right]}K_{1}^{\left[2\right]}a_{A}}{1+K_{1}^{\left[2\right]}a_{A}}$$

which is in quantitative agreement with the programmed reaction rate shown in Figure 9. Similar reductive methods have been developed to derive analytical approximations for enzyme catalysis in sinusoidally oscillated electric fields.^34^ A more robust methodology for the derivation of Equation 16 that applies to all programmed rate conditions, even those where simplifying assumptions cannot be made, is discussed in Section S7 of the supplemental information.

By allying this understanding with the LFE formalism in Equation 11, we determine the energetic requirements for optimal promotion via stepwise programs that simplifies to the rate function in Equation 16. Specifically, rate enhancement through the presented mechanism requires that (i) state [1] must regenerate a bare surface such that $K_{1}^{\left[1\right]}a_{A}\ll 1$ and (ii) state [2] (strong $BE_{B}$) must be more temporally efficient than state [1] (weak $BE_{B}$) at converting A to B∗. The latter condition is crucial; otherwise, state [1] would be better at executing *all* elementary steps and forced oscillation between state [1] and state [2] would decrease the rate of reaction. These conditions are formulated mathematically by requiring that the derivative of Equation 15 with respect to $BE_{B}$ evaluated at ${\text{BE}}_{B}^{\left[1\right]}$ is greater than zero for $K_{1}^{\left[1\right]}a_{A}\ll 1$ (Equation 17):

(Equation 17) $${\frac{d}{d\;BE_{B}}k_{2}K_{1}a_{A}|}_{BE_{B}^{\left[1\right]}}>0$$

Re-expression of this criterium in terms of LFE scaling parameters prescribes the explicit energetic requirements for promotion through stepwise programs (Equation 18):

(Equation 18) $$\omega_{2}<\omega_{-1}$$

corresponding to Regions I, IV, and V of Figure 7. Combination of Equation 18 with preceding analyses of quasi-static rate enhancement reveals that (i) Region IV is more effectively accelerated by quasi-static mechanisms rather than stepwise programs, (ii) Region V can be accelerated by either stepwise or quasi-static mechanism with an asymmetric waveform, and (iii) Region I is best suited to stepwise programs and can be further categorized into two distinct regimes, Region Ia and Ib which are separated by the condition $\omega_{2}=0$. The relevance of the $\omega_{2}=0$ condition to the kinetic behavior of Regions Ia and Ib, V is made clear by close examination of the consequences of the Sabatier principle on the maximum achievable time-averaged rates in these stepwise mechanism regimes.

**Figure 10:**
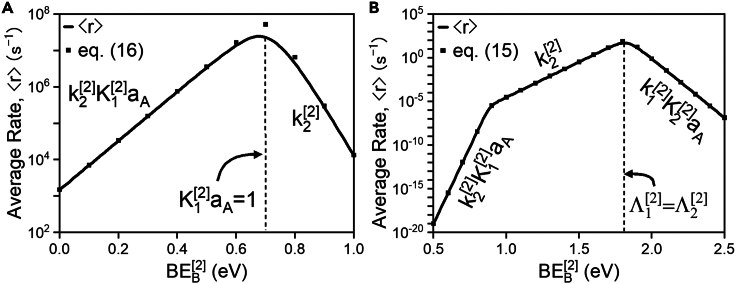
Volcano plots for stepwise mechanisms of Type I and V catalysts with $a_{A}=100,a_{B}=0,\delta =1.4\;\text{eV},T=373\;K\text{,}$$\delta t^{\left[2\right]}/\delta t^{\left[1\right]}=100$, and $BE_{B}^{\left[1\right]}=0\;\text{eV}$ (A) The volcano plot for stepwise mechanisms for Type Ib/V catalysts in Figure 7 with $\omega_{2}=1,\omega_{-1}=1.5$. (B) The volcano plot for stepwise mechanisms of Type Ia catalysts in Figure 7 with $\omega_{2}=-0.6$, $\omega_{-1}=2$. The optimal frequency was used with an initial guess at the center of the optimal frequency band, $f=\sqrt{\Lambda_{1}^{\left[2\right]}\Lambda_{2}^{\left[2\right]}}$.

Crucially, the derived energetic requirements (Equation 18) and the rates (Equation 15) of stepwise programs *do not* guarantee that ⟨r⟩ is greater than the Sabatier volcano maximum. The rate expression in Equation 15 is an exclusive function of rate and equilibrium constants in state [2] and, therefore, is subject to the constraints of the Sabatier principle for the energetic landscape of state [2]. From Equation 16, a transition occurs at $K_{1}^{\left[2\right]}a_{A}=1$, where $\left\langle r\right\rangle \approx k_{2}^{\left[2\right]}K_{1}^{\left[2\right]}a_{A}$ for $K_{1}^{\left[2\right]}a_{A}\ll 1$ and $\left\langle r\right\rangle \approx k_{2}^{\left[2\right]}$ when $K_{1}^{\left[2\right]}a_{A}\gg 1$. This transition alters the relationship between ⟨r⟩ and ${\text{BE}}_{B}^{\left[2\right]}$, of which both $k_{2}^{\left[2\right]}$ and $K_{1}^{\left[2\right]}$ are functions. Thus, Equation 16 implies a Sabatier-like dependence of ⟨r⟩ on ${\text{BE}}_{B}^{\left[2\right]}$, analogous to static catalysis, as illustrated in Figure 10A for a catalyst with $\omega_{2}=1$ and $\omega_{-1}=1.5$ (Region Ib).

Catalytic systems which lie in Region Ia are distinguished from those in Region Ib by the condition $\omega_{2}<0$, which implies that $k_{2}$ increases with increasing $BE_{B}$. The kinetic consequences of this distinct LFE scaling relationship are evident in Figure 10B which shows reaction rate as a function of ${\text{BE}}_{B}^{\left[2\right]}$ within Region Ia. In contrast to Figure 10A, the Sabatier maximum does not occur when $K_{1}^{\left[2\right]}a_{A}=1$, but instead at a much higher binding energy of B corresponding to the transition in the identity of the rate-controlling step from step 2 to step 1. Consequently, catalytic systems within Region Ia display three distinct kinetic regimes, each well-described by a simplified form of Equation 15. Each of these kinetic regimes is defined by a transition in the prevailing term in the denominator of Equation 15 which, in turn, quantifies a transition in the identity of the rate-determining step and most abundant surface intermediate. The kinetic consequences of the Sabatier principle on the stepwise mechanism of programmed catalysts are therefore entirely analogous to conventional static catalysis and can be quantitatively rationalized through familiar concepts grounded in fundamentals of heterogeneous catalysis kinetics.

**Figure 11:**
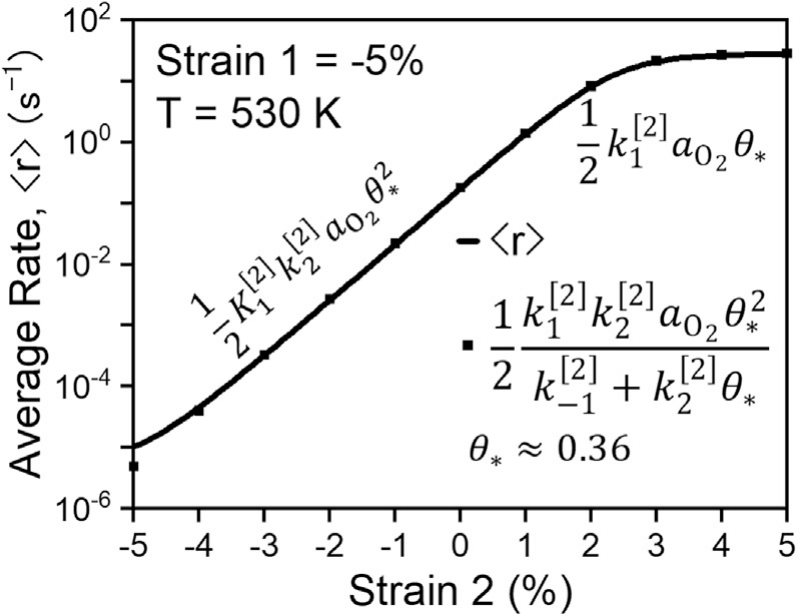
Numerically calculated (solid line) and analytically predicted (black squares) rate of catalytic CO oxidation as a function of strain in state [2] with an oscillation frequency in the center of the optimal frequency band, $f=\sqrt{k_{1}^{\left[2\right]}k_{2}^{\left[2\right]}a_{O_{2}}}$ The reaction rate is well described by a closed-form equation for pseudo-steady formation of O∗. At low/compressive strains, O_2_ sorption is quasi-equilibrated ($k_{-1}^{\left[2\right]}\gg k_{2}^{\left[2\right]}$), but at high/expansive strains, O_2_ adsorption becomes the rate-controlling step. Factors of ½ arise from non-optimization of the asymmetry of the square wave. Fractional coverages of CO∗ and O∗ are quasi-steady at ∼0.63 and ∼0.36, respectively.

We provide a final demonstration of the consequences of the Sabatier principle during programmed catalysis of an industrially relevant reaction by revisiting the case of catalytic CO oxidation on Pt (111) surfaces. In Figure 5, we showed that the promotional band of frequencies are bound by the characteristic pseudo-steady-state timescales for O∗ and O_2_∗ in the stretched lattice state (i.e., state [2]). That is to say that rate enhancement via the stepwise mechanism requires that the frequency of the forcing program is faster than the characteristic timescale of achieving PSS for O∗ but slower than that of achieving PSS for O_2_∗. Based on this understanding, we surmise that the rate of CO oxidation rates during stepwise programmed catalysis is readily described by the pseudo-steady rate of O∗ formation, with the PSS approximation faithfully applied to O_2_∗. From this insight, we derive a closed-form equation for reaction rate, shown in Figure 11, that both (i) quantitatively predicts ⟨r⟩ at minimal computational cost and (ii) captures the transition in rate control from O_2_∗ dissociation to O_2_ adsorption as a function of strain in state [2]. The quantitative accuracy and mechanistic clarity proffered by this rate function demonstrates that even complex, multi-step reaction sequences under programmed control can be studied and understood through conventional concepts of rate-controlling steps and PSS surface coverage native to static heterogeneous catalysis.

### Conclusions

Renewed interest in programmed, or dynamic, catalysis as an enabling technology for accelerating kinetically onerous reactions compels development of foundational mechanistic understanding to guide rational catalyst design and substrate selection. To address this need, this work develops mathematical tools that facilitate (i) calculation of programmed reaction rate, (ii) discovery of optimal forcing waveforms, or catalytic “programs”, and (iii) derivation of mechanistically interpretable rate expressions. From these advances, we identify the energetic requirements, or scaling relations, that permit rate enhancement via one of two distinct mechanisms—namely quasi-static and stepwise catalysis. The quasi-static mechanism, characterized by high forcing frequencies, effectively circumvents Sabatier limits to reaction rates, evidenced by optimal catalytic programs that switch between extrema in substrate binding affinity. In contrast, stepwise mechanisms, which operate at intermediate frequencies bounded by characteristic timescales of static catalysis, can increase the Sabatier limit relative to static catalysis, but not wholly circumvent it. The enduring consequences of the Sabatier principle for stepwise programmed catalysis are manifest in (i) persistent optimality of intermediate binding energies and (ii) time-averaged reaction rates that are dependent on rate constants from only one state of the catalytic program.

Stepwise programmed catalysis is so named because each kinetic state—each “instruction” of the catalytic program—executes a discrete component of the catalytic cycle. These kinetic states are tailored to their role in the program, and thus the potential energy surfaces and the time-widths are optimally chosen for their task. In this way, optimal forcing programs are rationalizable, and are likely to consist of a small number of kinetic states rather than a sinusoidal or more complex waveform. Further, this identification of tailored kinetic states suggest that amplitude and frequency are inappropriate descriptors of forcing programs, and instead the precise potential energy surfaces and state durations are the salient parameters.

In both mechanisms, we identify simplifying conditions that facilitate derivation of Langmuir-Hinshelwood rate functions for the programmed reaction rate. Construal of programmed catalysis in these familiar mathematical forms enables precise mechanistic interpretation of rate-controlling steps and most abundant surface intermediates—identically to conventional steady-state heterogeneous catalysis.

### Limitations of the study

Numerical calculation of limit cycles are sensitive to floating point errors and may require careful vetting to ensure the solution is sensible. Many of the analyses discussed in this work are specifically applicable to the linear three-step reaction sequence, but the approaches are generalizable to more complex reaction networks.

## STAR★Methods

### Key resources table

**Table:** 

REAGENT or RESOURCE	SOURCE	IDENTIFIER
**Software and algorithms**		

Matlab R2020a	MathWorks	https://www.mathworks.com/
Matlab codes for calculating reaction rates and finding optimal waveforms	This paper	https://zenodo.org/records/10059273

### Resource availability

#### Lead contact

• Further information and requests for code may be directed to the lead contact, Brandon Foley (foley26@llnl.gov).

#### Materials availability

• This study did not generate any new reagents.

#### Data and code availability

• All data reported in this paper will be shared by the lead contact upon request.
• All original code is provided in of the supplemental information and are publicly available as of the date of publication from zenodo.org at https://doi.org/10.5281/zenodo.10059273.
• Any additional information required to reanalyze the data reported in this paper is available from the lead contact upon request.

### Method details

All functions and scripts are written in Octave GNU and Matlab ® 2020a. The code is provided in Section S1 of the supplemental information and are available to download for free from zenodo.org at https://doi.org/10.5281/zenodo.10059273. Computational times are measured using the “tic” and “toc” functions. The reaction kinetics for Scheme 2 in the “illustrative case studies of programmable catalysis” section are identical to those reported by Ardagh et al.,^31^ which included bounds on the values for some of the intrinsic rate constants which breaks down the linearity of some of the free energy scaling relationships. In “mechanistic underpinnings of rate enhancement during programmable catalysis” section, these bounds are removed to maintain linear relationships over the entire free energy landscape.
