# Supplementary material for: Clarifying mechanisms and kinetics of programmable catalysis
Source: iScience. 2024 Mar 20;27(4):109543. doi: 10.1016/j.isci.2024.109543 (PMC11024910; doi:10.1016/j.isci.2024.109543)
Supplement: Document S1. Figures S1–S5 and Tables S1–S3 [file mmc1.pdf]

iScience, Volume *27*

Supplemental information

Clarifying mechanisms and kinetics of programmable catalysis

Brandon L. Foley and Neil K. Razdan

Contents

Section S1. Matlab code for calculating limit cycles and finding optimal waveform

Section S1.1. Matlab code for calculating limit cycles for the three step-reaction scheme with discretization of continuous the kinetic oscillation

Section S1.2. Matlab code for calculating limit cycles for general reaction scheme

Section S1.3. Finding optimal wave shape and frequency for a reaction

Section S2. Derivation of the time-averaged reaction rate (eq. 3)

Section S3. General solution to periodically forced linear system of chemical reactions.

Section S4. Finding limit cycle solutions for non-linear reaction systems

Section S5. Calculation times and limit cycles for varying number of steps in sine wave approximation

Section S6. CO oxidation Matlab codes

Section S6.1. CO oxidation rate constants

Section S6.2. Calculation of limit cycles

Section S7. Calculation of weighted-average rate constants

References

Section S1. Matlab code for calculating limit cycles and finding optimal waveform

Section S1.1. Matlab code for calculating limit cycles for the three step-reaction scheme with discretization of continuous the kinetic oscillation

The code detailed below shows an example of calculating the limit cycles for the three-step reaction scheme $A + * \rightleftarrows A^*$, $A^* \rightleftarrows B^*$, and $B^* \rightleftarrows B + *$ using the kinetics from Ardagh et al. This code includes a for loop for calculating the limit cycles as a function of wavelength (inverse frequency) and storing the results in the array "savedata" for access later. While this code uses the three-step reaction scheme as an example, by changing the input cell k, one can control simultaneously both the number of reactions and the number of discretization steps of the oscillation. The number of arrays in cell k gives the number of discretization steps (or kinetic states), and the width of these arrays gives the number of reaction steps. The arrays in cell k always have two rows—the first row is populated with the forward rate constants and the second row is populated with reverse rate constants. This code assumes that the reaction steps are in series, but could be modified to consider linear reaction schemes with parallel reactions by modification of the arrays in cell A.

```
%This script finds the time-averaged rate during the limit cycle for an
%n-step reaction oscillating between m kinetic states. The kinetics for a
%three-step reaction has been implemented.

%k{j} is k's for state j
%row 1 is kf, row 2 is kr
%you can specify any number of reactions and states
clear;tic
inc=1; % counter
inc2=0; %another counter

%% Parameters
Tc = 100; % deg C
BEAeV0 = 1.4; % eV
delta = 1.4; % eV
gamma = 0.5; % dimensionless

% Lower and Upper change in BE
UL = (0.1-delta)*2; %eV
UH = (1.03-delta)*2; % eV

%choose how many steps in your square wave
NoSteps=2;

%what wavelengths will be tested
wavespan=logspace(-10,10,101);

%initialize vector
savedata{1}=zeros(length(wavespan),2);

%Choose which waveform
BEfun=@(t) (UH-UL)/2*cos(t*(2*pi))+UH-(UH-UL)/2; %sinewave or square wave (for NoSteps=2)
%BEfun=@(t) (UH-UL)/2*sawtooth(t*2*pi(),0.5)+UH-(UH-UL)/2;; % triangle wave
%BEfun=@(t) (UH-UL)/2*sawtooth(0.01,t)+UH-(UH-UL)/2; %sawtooth
%BEfun=@(t) (UH-UL)/2*sawtooth(t*2*pi(),0.9)+UH-(UH-UL)/2; %reverse sawtooth
%BEfun=@(t) (UH-UL)/2*rand()+UH-(UH-UL)/2;

ts=linspace(0,1,NoSteps+1);

%choose the discretization -- greater NoSteps = longer comp time (~ NoSteps^2)
```

```
%2 with sinewave gives squarewave; 100-400 seems like enough steps
    inc=1;
    inc2=inc2+1;
% BEP relationship parameters
alpha = 0.6; % dimensionless
beta = 102e3; % J/gmol

% Fluid phase pressures
Pa = 98; % bar
Pb = 2; % bar

%% Dauenhauer rate constants
% Temperature
T = Tc + 273.15; % K

% Binding energies


% Overall reaction enthalpy
delHovr = 0; % J/gmol

%find k's for each kinetic state -- this is for a three-step reaction
%example but can be changed for any reaction
for j=1:NoSteps

    BEB0 = delta*96.485e3 + delHovr; % J/mol
    BEA0 = (BEB0 - ((1-gamma)*delta*96.485e3) - (delHovr))/gamma; % J/gmol
    deltE0 = BEfun(ts(j))*96.485e3; % J/mol

    % Binding energy (J/gmol)
    BEa = BEA0 + deltE0;
    BEb = BEB0 + gamma*deltE0;

    % Heat of reaction (J/gmol)
    delH1 = -BEa; % A --> A*
    delH2 = delHovr + BEa - BEb; % A* --> B*
    delH3 = BEb; % B* --> B

    % E - Activation energy in the Arrhenius Equation (J/gmol)
    R = 8.314459848; % J/gmol-K
    Eaf1 = 0e3;
    Eaf2 = beta + alpha*delH2;
    Eaf3 = delH3;

    Eaf1 = max(0,Eaf1);
    Eaf2 = max(0,Eaf2);
    Eaf3 = max(0,Eaf3);

    % Pre-exponential factor
    Af1 = 1e6; % 1/bar-sec
    Af2 = 1e13; % 1/sec
    Af3 = 1e13; % 1/sec

    % Equilibrium constants
    K1 = 1e-7*exp(-delH1/(R*T)); % 1/bar
    K2 = 1*exp(-delH2/(R*T)); % unitless
```

```
    K3 = 1e7*exp(-delH3/(R*T)); % bar

    % Rate constants
        k{j}(1,:) = [Af1*exp(-Eaf1/(R*T)), Af2*exp(-Eaf2/(R*T)), Af3*exp(-Eaf3/(R*T))];
        k{j}(2,:)=k{j}(1,:)./[K1,K2,K3];
        k{j}(1,1)=k{j}(1,1)*Pa;
        k{j}(2,3)=k{j}(2,3)*Pb;
        k{j}(2,1)=min(1e25,k{j}(2,1)); % Capping k-1 so matrices are not poorly scaled
end

NoRxns=size(k{1},2);
NoStates=max(size(k));
NoSpecies=NoRxns-1;

%initialize coefficient matrix A
for j=1:NoStates
  A{j}=zeros(NoRxns);
end

Amat=zeros(2*(NoRxns-1)*NoStates);
bvec=zeros(2*(NoRxns-1)*NoStates,1);

for wavelength=wavespan %vary the wavelength (1/frequency)

%even discretization in time
    d(1:NoSteps)=wavelength/NoSteps;

%fill coefficient matrix A
for m=1:NoStates
  for j=2:NoRxns-1
    A{m}(j,j-1)=k{m}(1,j-1);
    A{m}(j,j)=-k{m}(1,j)-k{m}(2,j-1);
    A{m}(j,j+1)=k{m}(2,j);
  end
  A{m}(1,1)=-k{m}(1,1)-k{m}(2,NoRxns);
  A{m}(1,2)=k{m}(2,1);
  A{m}(1,NoRxns)=A{m}(1,NoRxns)+k{m}(1,NoRxns);

  A{m}(NoRxns,NoRxns-1)=A{m}(NoRxns,NoRxns-1)+k{m}(1,NoRxns-1);



  A{m}(NoRxns,NoRxns)=A{m}(NoRxns,NoRxns)-k{m}(1,NoRxns)-k{m}(2,NoRxns-1);
  A{m}(NoRxns,1)=A{m}(NoRxns,1)+k{m}(2,NoRxns);

  %store column 2 of matrix A as b
  b{m}=A{m}(:,2);

  %subtract b from each column in A
  for n=1:size(A{m},2)
    A{m}(:,n)=A{m}(:,n)-b{m};
  end

  %delete the 2nd column and row of A and b
  A{m}(2,:)=[];
  A{m}(:,2)=[];
```

```
  b{m}(2)=[];

  %find the particular solution
  p{m}=-A{m}\b{m};

  %find the eigenvalues and eigenvectors of A'
  [V{m},D{m}]=eig(A{m});


%fill matrices for solving for coefficients and species at each boundary
  for n=1:NoSpecies
    Amat(NoSpecies*(m-1)+n,NoSpecies*(m-1)+n)=-1;
    Amat(NoSpecies*(m-1)+n,NoSpecies*(NoStates+m-1)+1:NoSpecies*(NoStates+m))=V{m}(n,:);
    if m<NoStates
      Amat(NoSpecies*NoStates+NoSpecies*(m-1)+n,NoSpecies*m+n)=-1;
    else
      Amat(NoSpecies*NoStates+NoSpecies*(m-1)+n,n)=-1;
    end
    Amat(NoSpecies*NoStates+NoSpecies*(m-1)+n,NoSpecies*(NoStates+m-
1)+1:NoSpecies*(NoStates+m))=V{m}(n,:).*exp(diag(D{m}*d(m)))';
    bvec(NoSpecies*(m-1)+n,1)=p{m}(n);
    bvec(NoSpecies*NoStates+NoSpecies*(m-1)+n)=p{m}(n);
  end
end
%solve the linear equations to find c and thetas at the boundary
x=-Amat\bvec;

%extract c's from the vector x and store as c{j}
for j=1:NoStates
  c{j}=x(NoSpecies*(NoStates+j-1)+1:(NoSpecies)*(NoStates+j));
end

rate=0;
%calculate the rate by evaluating the analytical solution for the
%time-averaged rate
if NoSpecies==1
  for j=1:NoStates
    rate=rate+sum(k{j}(1,NoRxns)*(1*d(j)-c{j}.*V{j}(NoSpecies,:)'./diag(D{j}).*(exp(diag(D{j})*d(j))-1)-
p{j}(NoSpecies)*d(j)));
    rate=rate-sum(k{j}(2,NoRxns)*(c{j}.*V{j}(NoSpecies,:)'./diag(D{j}).*(exp(diag(D{j})*d(j))-
1)+p{j}(NoSpecies)*d(j)));
  end
else
  for j=1:NoStates
    rate=rate+k{j}(1,NoRxns)*(sum(-c{j}.*V{j}(NoSpecies,:)'./diag(D{j}).*(1-
exp(diag(D{j}*d(j)))))+p{j}(NoSpecies)*d(j));
    rate=rate-k{j}(2,NoRxns)*(sum(-c{j}.*V{j}(1,:)'./diag(D{j}).*(1-exp(diag(D{j}*d(j)))))+p{j}(1)*d(j));
  end
end
 %re-initialize A
for m=1:NoStates
    A{m}=[A{m}(:,1),zeros(size(A{m},1),1),A{m}(:,2:end)];
    A{m}=[A{m}(1,:);zeros(1,size(A{m},2));A{m}(2:end,:)]*0;
  end
rate=rate/(sum(d));
```

```
savedata{inc2}(inc,:)=[wavelength,rate];

inc=inc+1;
end
toc
```

Section S1.2. Matlab code for calculating limit cycles for general reaction scheme

The code copied below is the same principle as the code in Section S1.1 but does not include the kinetics from Ardagh et al. or the discretization code to better illustrate how to include multiple kinetic states and vary the number of reactions. This code is also easy to vary the asymmetry of the kinetic states. As set-up in this example, the code considers two kinetic states with three reaction steps in series, and varies the time in each kinetic state.

```
clear
%k{j} is k's for kinetic state j
%row 1 is kf, row 2 is kr

gg=0;
%specify the two kinetic states
%activities of species are lumped into the rate constants, i.e., k1(in
%code) = k1(real)*aA.
k{1}=[98000000,1805883084.44119,446067282883.190;1.00000000000000e+19,4.99913022560784e-09,2000000];
k{2}=[98000000,52.5222955208898,0.122694139474606;12191.0874650912,0.000528597458645033,2000000];
times=zeros(1,100);

n=logspace(-15,0,101);
m=n;
[N,M]=meshgrid(n,m);
R=N;
%for loops to vary the times in each kinetic state
for nn=1:size(N,1)
  for mm=1:size(N,2)
d(1)=N(nn,mm);
d(2)=M(nn,mm);

NoRxns=size(k{1},2);
NoStates=max(size(k));
NoSpecies=NoRxns-1;
Amat=zeros(2*(NoRxns-1)*NoStates);
bvec=zeros(2*(NoRxns-1)*NoStates,1);
%initialize arrays
for j=1:NoStates
  A{j}=zeros(NoRxns);
end


for m=1:NoStates
    %populate matrix A, assumes linear rxn scheme with no parallel reactions
  for j=2:NoRxns-1
    A{m}(j,j-1)=k{m}(1,j-1);
    A{m}(j,j)=-k{m}(1,j)-k{m}(2,j-1);
    A{m}(j,j+1)=k{m}(2,j);
```

```
  end
  %convert system of odes into a solvable, non-singular system
  A{m}(1,1)=-k{m}(1,1)-k{m}(2,NoRxns);
  A{m}(1,2)=k{m}(2,1);
  A{m}(1,NoRxns)=A{m}(1,NoRxns)+k{m}(1,NoRxns);

  A{m}(NoRxns,NoRxns-1)=A{m}(NoRxns,NoRxns-1)+k{m}(1,NoRxns-1);
  A{m}(NoRxns,NoRxns)=A{m}(NoRxns,NoRxns)-k{m}(1,NoRxns)-k{m}(2,NoRxns-1);
  A{m}(NoRxns,1)=A{m}(NoRxns,1)+k{m}(2,NoRxns);

  b{m}=A{m}(:,2);

  for n=1:size(A{m},2)
    A{m}(:,n)=A{m}(:,n)-b{m};
  end
  A{m}(2,:)=[];
  A{m}(:,2)=[];
  b{m}(2)=[];

  %solve particular solutions
  p{m}=-A{m}\b{m};
  %get eigenvalues and eigenvectors
  [V{m},D{m}]=eig(A{m});

  %populate system of equations Amat*x=bvec where x is vector of arbitrary
  %constants and fractional coverages at BC's
  for n=1:NoSpecies
    Amat(NoSpecies*(m-1)+n,NoSpecies*(m-1)+n)=-1;
    Amat(NoSpecies*(m-1)+n,NoSpecies*(NoStates+m-1)+1:NoSpecies*(NoStates+m))=V{m}(n,:);
    if m<NoStates
      Amat(NoSpecies*NoStates+NoSpecies*(m-1)+n,NoSpecies*m+n)=-1;
    else
      Amat(NoSpecies*NoStates+NoSpecies*(m-1)+n,n)=-1;
    end
    Amat(NoSpecies*NoStates+NoSpecies*(m-1)+n,NoSpecies*(NoStates+m-
1)+1:NoSpecies*(NoStates+m))=V{m}(n,:).*exp(diag(D{m}*d(m)))';
    bvec(NoSpecies*(m-1)+n,1)=p{m}(n);
    bvec(NoSpecies*NoStates+NoSpecies*(m-1)+n)=p{m}(n);
    end
end
    %solve systems of equations for constants and fractional coverages at
    %BC's
x=-Amat\bvec;
%extract arbitrary constants from vector x
for j=1:NoStates
  c{j}=x(NoSpecies*(NoStates+j-1)+1:(NoSpecies)*(NoStates+j));
end

%numerically evaluate rate
rate=0;
if NoSpecies==1
  for j=1:NoStates
    rate=rate+sum(k{j}(1,NoRxns)*(1*d(j)-c{j}.*V{j}(NoSpecies,:)'./diag(D{j}).*(exp(diag(D{j})*d(j))-1)-
p{j}(NoSpecies)*d(j)));
    rate=rate-sum(k{j}(2,NoRxns)*(c{j}.*V{j}(NoSpecies,:)'./diag(D{j}).*(exp(diag(D{j})*d(j))-
1)+p{j}(NoSpecies)*d(j)));
```

```
  end
else
  for j=1:NoStates
    rate=rate+k{j}(1,NoRxns)*(sum(-c{j}.*V{j}(NoSpecies,:)'./diag(D{j}).*(1-
exp(diag(D{j}*d(j)))))+p{j}(NoSpecies)*d(j));
    rate=rate-k{j}(2,NoRxns)*(sum(-c{j}.*V{j}(1,:)'./diag(D{j}).*(1-exp(diag(D{j}*d(j)))))+p{j}(1)*d(j));
  end
end
rate=rate/(sum(d));
R(nn,mm)=rate;
  end;end
%plot
figure;surf(N,M,R)
set(gca,'xscale','log');set(gca,'yscale','log')
view(2)
shading interp
xlabel('\deltat^{[1]}');ylabel('\deltat^{[2]}');colormap jet;c=colorbar;c.Label.String='<r> / s^{-1}';
```

Section S1.3. Finding optimal wave shape and frequency for a reaction

The code for finding the optimal waveshape and wavelength are detailed below in two parts. First, the rate code is written as a function. The solver part is the same as those detailed in Section S1, but the inputs to this function are energies that will be oscillated between, and relative size of each step, and the wavelength of the kinetic oscillation. The output is the reaction rate. As a solver we use the built-in Matlab ® function fminunc, which is for *unconstrained* optimization problems. We want a constrained optimization, so we artificially constrain the values of energies (which we restrict to a range between specified lower and upper bound values) and dsizes (which are positive values) and wavelength (also a positive value). The energies are constrained by defining a logistic function which equals the lowerbound at $-\infty$ and the upperbound at $+\infty$. The “energies” vector that is used as input are the points at which this logisitic function is evaluated. The dsizes vector input is natural log of the relative sizes of each steps. The size of energies and dsizes must match and are equal to the number of kinetic states in the oscillation. An example script for running the optimization is copied below. The fminunc requires the optimization toolbox in Matlab ®, but is available for free in Octave.

Script

```
options = optimset('PlotFcns',@optimplotfval,'MaxIter',1e5,'MaxFunEvals',1e5, 'TolFun',1e-100,'TolX',1e-
100);

[a,b]=fminunc(@(x) -threesteprxnrates(x(1:2),x(3:4),10^x(5)), [-1,1,0,0,-6],options)
```

Function

```
function [rate]=threesteprxnrates(energies,dsizes,wavelength)
%energies is where a logistic function is evaluated, -infinity for energies
%gives the lowerbound (0.1 eV for BEb), +infinity gives the upperbound
%(1.03 BEb).

%dsizes is the ln(relative size) of each step

%beta version
%k{j} is k's for state j
%row 1 is kf, row 2 is kr
%you can specify any number of reactions and states, but I'm not positive it will work correctly
dsizes=exp(dsizes);
dsizes=dsizes./sum(dsizes);
delta=1.4;
```

```
% Lower and Upper change in BE
%artifical upper and lowerbounds of 0.1 and 1.03 eV for BEb
UL = (0.1-delta)*2; %eV
UH = (1.03-delta)*2; % eV

%logistic function forces upper and lower bound constraints to be satisfied
energies=(2./(1+exp(-energies))-1)*(UH-UL)/2+UH-(UH-UL)/2;

NoSteps=length(energies);

    %inc2=inc2+1;

%% Parameters
Tc = 100; % deg C
BEAeV0 = 1.4; % eV
delta = 1.4; % eV
gamma = 0.5; % dimensionless

% BEP relationship parameters
alpha = 0.6; % dimensionless
beta = 102e3; % J/gmol

% Fluid phase pressures
Pa = 98; % bar
Pb = 2; % bar

%% Dauenhauer rate constants
% Temperature
T = Tc + 273.15; % K

% Binding energies


% Overall reaction enthalpy
delHovr = 0; % J/gmol

for j=1:NoSteps
    BEB0 = delta*96.485e3 + delHovr; % J/mol
    BEA0 = (BEB0 - ((1-gamma)*delta*96.485e3) - (delHovr))/gamma; % J/gmol
    deltE0 = energies(j)*96.485e3; % J/mol

    % Binding energy (J/gmol)
    BEa = BEA0 + deltE0;
    BEb = BEB0 + gamma*deltE0;

    % Heat of reaction (J/gmol)
    delH1 = -BEa; % A --> A*
    delH2 = delHovr + BEa - BEb; % A* --> B*
    delH3 = BEb; % B* --> B

    % E - Activation energy in the Arrhenius Equation (J/gmol)
    R = 8.314459848; % J/gmol-K
    Eaf1 = 0e3;
    Eaf2 = beta + alpha*delH2;
    Eaf3 = delH3;
```

```
    Eaf1 = max(0,Eaf1);
    Eaf2 = max(0,Eaf2);
    Eaf3 = max(0,Eaf3);

    % Pre-exponential factor
    Af1 = 1e6; % 1/bar-sec
    Af2 = 1e13; % 1/sec
    Af3 = 1e13; % 1/sec

    % Equilibrium constants
    K1 = 1e-7*exp(-delH1/(R*T)); % 1/bar
    K2 = 1*exp(-delH2/(R*T)); % unitless
    K3 = 1e7*exp(-delH3/(R*T)); % bar

    % Rate constants
        k{j}(1,:) = [Af1*exp(-Eaf1/(R*T)), Af2*exp(-Eaf2/(R*T)), Af3*exp(-Eaf3/(R*T))];
        k{j}(2,:)=k{j}(1,:)./[K1,K2,K3];
        k{j}(1,1)=k{j}(1,1)*Pa;
        k{j}(2,3)=k{j}(2,3)*Pb;
        k{j}(2,1)=min(1e25,k{j}(2,1));
end

NoRxns=size(k{1},2);
NoStates=max(size(k));
NoSpecies=NoRxns-1;

for j=1:NoStates
  A{j}=zeros(NoRxns);
end

for m=1:NoStates
  for j=2:NoRxns-1
    A{m}(j,j-1)=k{m}(1,j-1);
    A{m}(j,j)=-k{m}(1,j)-k{m}(2,j-1);
    A{m}(j,j+1)=k{m}(2,j);
  end
  A{m}(1,1)=-k{m}(1,1)-k{m}(2,NoRxns);
  A{m}(1,2)=k{m}(2,1);
  A{m}(1,NoRxns)=A{m}(1,NoRxns)+k{m}(1,NoRxns);

  A{m}(NoRxns,NoRxns-1)=A{m}(NoRxns,NoRxns-1)+k{m}(1,NoRxns-1);
  A{m}(NoRxns,NoRxns)=A{m}(NoRxns,NoRxns)-k{m}(1,NoRxns)-k{m}(2,NoRxns-1);
  A{m}(NoRxns,1)=A{m}(NoRxns,1)+k{m}(2,NoRxns);

  b{m}=A{m}(:,2);

  for n=1:size(A{m},2)
    A{m}(:,n)=A{m}(:,n)-b{m};
  end
  A{m}(2,:)=[];
  A{m}(:,2)=[];
  b{m}(2)=[];

  p{m}=-A{m}\b{m};
  [V{m},D{m}]=eig(A{m});
end
```

```
    Amat=zeros(2*(NoRxns-1)*NoStates);
bvec=zeros(2*(NoRxns-1)*NoStates,1);
d(1:NoSteps)=wavelength.*dsizes;

for m=1:NoStates
  for n=1:NoSpecies
    Amat(NoSpecies*(m-1)+n,NoSpecies*(m-1)+n)=-1;
    Amat(NoSpecies*(m-1)+n,NoSpecies*(NoStates+m-1)+1:NoSpecies*(NoStates+m))=V{m}(n,:);
    if m<NoStates
      Amat(NoSpecies*NoStates+NoSpecies*(m-1)+n,NoSpecies*m+n)=-1;
    else
      Amat(NoSpecies*NoStates+NoSpecies*(m-1)+n,n)=-1;
    end
    Amat(NoSpecies*NoStates+NoSpecies*(m-1)+n,NoSpecies*(NoStates+m-
1)+1:NoSpecies*(NoStates+m))=V{m}(n,:).*exp(diag(D{m}*d(m)))';
    bvec(NoSpecies*(m-1)+n,1)=p{m}(n);
    bvec(NoSpecies*NoStates+NoSpecies*(m-1)+n)=p{m}(n);
  end
end
x=-Amat\bvec;
for j=1:NoStates
  c{j}=x(NoSpecies*(NoStates+j-1)+1:(NoSpecies)*(NoStates+j));
end


rate=0;
  for j=1:NoStates
    rate=rate+k{j}(1,NoRxns)*(sum(-c{j}.*V{j}(NoSpecies,:)'./diag(D{j}).*(1-
exp(diag(D{j}*d(j)))))+p{j}(NoSpecies)*d(j));
    rate=rate-k{j}(2,NoRxns)*(sum(-c{j}.*V{j}(1,:)'./diag(D{j}).*(1-exp(diag(D{j}*d(j)))))+p{j}(1)*d(j));
  end
rate2=1/sum(d)*(k{1}(1,3)*(c{1}(1)*V{1}(2,1)/-D{1}(1,1)*(1-exp(D{1}(1,1)*d(1)))+c{1}(2)*V{1}(2,2)/-
D{1}(2,2)*(1-exp(D{1}(2,2)*d(1)))+p{1}(2)*d(1))-k{1}(2,3)*(c{1}(1)*V{1}(1,1)/-D{1}(1,1)*(1-
exp(D{1}(1,1)*d(1)))+c{1}(2)*V{1}(1,2)/-D{1}(2,2)*(1-
exp(D{1}(2,2)*d(1)))+p{1}(1)*d(1))+k{2}(1,3)*(c{2}(1)*V{2}(2,1)/-D{2}(1,1)*(1-
exp(D{2}(1,1)*d(2)))+c{2}(2)*V{2}(2,2)/-D{2}(2,2)*(1-exp(D{2}(2,2)*d(2)))+p{2}(2)*d(2))-
k{2}(2,3)*(c{2}(1)*V{2}(1,1)/-D{2}(1,1)*(1-exp(D{2}(1,1)*d(2)))+c{2}(2)*V{2}(1,2)/-D{2}(2,2)*(1-
exp(D{2}(2,2)*d(2)))+p{2}(1)*d(2)));
 rate=rate/(sum(d));
```

Section S2. Derivation of the time-averaged reaction rate (eq. 3)

Equation (S1) describes the time-evolution of the fractional coverage of I* with time-dependent rate constants:

$$\frac{\mathrm{d}\theta_{\mathrm{I}^*}}{\mathrm{d}t} = -k_2(t)\theta_{\mathrm{I}^*} + k_1(t)a_{\mathrm{A}}(1-\theta_{\mathrm{I}^*}) \tag{S1}$$

This differential equation can be separated piecewise into regions of time where the rate constants are constant (eq. (S2)):

$$\frac{\mathrm{d}\theta_{\mathrm{I}^*}}{\mathrm{d}t} = -(k_2^{[j]} + k_1^{[j]}a_{\mathrm{A}})\theta_{\mathrm{I}^*} + k_1^{[j]}a_{\mathrm{A}} \tag{S2}$$

which has an analytical solution given by eq. (S3):

$$\theta_{\mathrm{I}^*}^{[j]} = c_j \exp\left(-\left(k_2^{[j]} + k_1^{[j]} a_A\right) t\right) + \frac{k_1^{[j]} a_{\mathrm{A}}}{\left(k_2^{[j]} + k_1^{[j]} a_A\right)} \ \forall\ t \in t^{[j]} \tag{S3}$$

where $t = 0$ is the start of each kinetic state. The integration constants c_j are found by enforcing a periodic boundary condition that $\theta_{I^*}^{[1]}(t = 0) = \theta_{I^*}^{[2]}(t = \delta t^{[2]})$ and $\theta_{I^*}^{[2]}(t = 0) = \theta_{I^*}^{[1]}\left(t = \delta t^{[1]}\right)$ (eq. (S4)):

$$\begin{gathered} c_1 + \frac{k_1^{[1]} a_{\mathrm{A}}}{\left(k_2^{[1]} + k_1^{[1]} a_A\right)} = c_2 \exp\left(-\left(k_2^{[2]} + k_1^{[2]} a_A\right)\delta t^{[2]}\right) + \frac{k_1^{[2]} a_{\mathrm{A}}}{\left(k_2^{[2]} + k_1^{[2]} a_A\right)} \\ c_2 + \frac{k_1^{[2]} a_{\mathrm{A}}}{\left(k_2^{[2]} + k_1^{[2]} a_A\right)} = c_1 \exp\left(-\left(k_2^{[1]} + k_1^{[1]} a_A\right)\delta t^{[1]}\right) + \frac{k_1^{[1]} a_{\mathrm{A}}}{\left(k_2^{[1]} + k_1^{[1]} a_A\right)} \end{gathered} \tag{S4}$$

which is a linear equation with the solution given by eq. (S5):

$$\begin{gathered} c_1 = \frac{\exp\left(-k_2^{[2]} \delta t^{[2]}\right) - 1}{1 - \exp\left(-k_1^{[1]} a_A \delta t^{[1]} - k_2^{[2]} \delta t^{[2]}\right)} \\ c_2 = \frac{1 - \exp\left(-k_1^{[1]} a_{\mathrm{A}} \delta t^{[1]}\right)}{1 - \exp\left(-k_1^{[1]} a_A \delta t^{[1]} - k_2^{[2]} \delta t^{[2]}\right)} \end{gathered} \tag{S5}$$

The time-averaged rate is found by taking an integra of the rate over a single oscillation and dividing by the wavelength, which gives eq. (S6) and is equivalent to eq. (3).

$$\begin{aligned} \langle r \rangle &= \frac{1}{(\delta t^{[1]} + \delta t^{[2]})} \int_0^{\delta t^{[1]} + \delta t^{[2]}} k_2\ (t) \theta_{\mathrm{I}^*}\ (\mathrm{t}) \mathrm{d}t = \frac{1}{(\delta t^{[1]} + \delta t^{[2]})} \int_0^{\delta t^{[2]}} k_2^{[2]} \theta_{\mathrm{I}^*}^{[2]} \mathrm{d}t \\ &= \frac{\left(1 - \exp\left(-k_2^{[2]} t\right)\right) (1 - \exp\left(-k_1^{[1]} a_{\mathrm{A}} \delta t^{[1]}\right)}{(\delta t^{[1]} + \delta t^{[2]}) \left(1 - \exp\left(-k_1^{[1]} a_A \delta t^{[1]} - k_2^{[2]} \delta t^{[2]}\right)\right)} \end{aligned} \tag{S6}$$

Section S3. General solution to periodically forced linear system of chemical reactions.

The coupled ordinary differential equations (ODEs) for any linear reaction scheme can be written in the vector form given by eq. (S7).

$$\frac{d\boldsymbol{\theta}}{dt} = \boldsymbol{A}\boldsymbol{\theta} + \boldsymbol{b} \tag{S7}$$

This system of ODEs has a general solution over each kinetic state given by eq. (S8):

$$\boldsymbol{\theta}(t) = \boldsymbol{V}^{[j]} \exp\left(\boldsymbol{\Lambda}^{[j]} t\right) \boldsymbol{c}^{[j]} + \boldsymbol{p}^{[j]} \ \text{ for } t \in \boldsymbol{t}^{[j]} \tag{S8}$$

where $\boldsymbol{p}^{[j]}$ is the particular, or steady-state, solution for kinetic state [j] given by eq. (S9):

$$\boldsymbol{p}^{[j]} = -\left(\boldsymbol{A}^{[j]}\right)^{-1} \boldsymbol{b}^{[j]} \tag{S9}$$

$\boldsymbol{\Lambda}^{[j]}$ is the diagonal square matrix of eigenvalues of $\boldsymbol{A}^{[j]}$ (eq. (S10))**:**

$$\mathbf{\Lambda}^{[j]} = \begin{bmatrix} \Lambda_1^{[j]} & 0 & \cdots & 0 \\ 0 & \Lambda_2^{[j]} & \cdots & 0 \\ \vdots & \vdots & \ddots & \vdots \\ 0 & 0 & \cdots & \Lambda_n^{[j]} \end{bmatrix} \tag{S10}$$

$\boldsymbol{V}^{[j]}$ is the square matrix of eigenvectors of $\boldsymbol{A}^{[j]}$ (eq. (S11)):

$$\boldsymbol{V}^{[j]} = \left[v_1^{[j]} \quad \ldots \quad v_n^{[j]} \right] \tag{S11}$$

The integration constants are found by solving the periodic and continuity boundary conditions of the form given by eq. (S12):

$$\boldsymbol{V}^{[j]} \exp\left(\mathbf{\Lambda}^{[j]} \delta t^{[j]}\right) \boldsymbol{c}^{[j]} - \boldsymbol{V}^{[j+1]} \boldsymbol{c}^{[j+!]} = \boldsymbol{p}^{[j+!]} - \boldsymbol{p}^{[j]} \tag{S12}$$

These equations can be written in the form of a single linear matrix equation (eq. (S13)):

$$\underbrace{\begin{bmatrix} & & & \vdots & & & \\ 0 & \cdots & \boldsymbol{V}^{[j]} \exp\left(\mathbf{\Lambda}^{[j]} \delta t^{[j]}\right) & -\boldsymbol{V}^{[j+1]} & \cdots & 0 & \\ 0 & \cdots & \cdots & \boldsymbol{V}^{[j+1]} \exp\left(\mathbf{\Lambda}^{[j+1]} \delta t^{[j+1]}\right) & -\boldsymbol{V}^{[j+2]} & \cdots \\ & & & \vdots & & & \end{bmatrix}}_{\boldsymbol{M}} \underbrace{\begin{bmatrix} \boldsymbol{c}^{[1]} \\ \vdots \\ \boldsymbol{c}^{[j]} \\ \boldsymbol{c}^{[j+!]} \\ \vdots \\ \boldsymbol{c}^{[n]} \end{bmatrix}}_{\boldsymbol{c}} = \underbrace{\begin{bmatrix} \vdots \\ \boldsymbol{p}^{[j+!]} - \boldsymbol{p}^{[j]} \\ \boldsymbol{p}^{[j+2]} - \boldsymbol{p}^{[j+1]} \\ \vdots \end{bmatrix}}_{\boldsymbol{q}} \tag{S13}$$

Equation (S13) is easily solved to find the integration constants (eq. (S14)).

$$\boldsymbol{c} = \boldsymbol{M}^{-1} \boldsymbol{q} \tag{S14}$$

Section S4. Finding limit cycle solutions for non-linear reaction systems

The dynamic steady-state with periodicity λ is the solution to systems of differential equations where the periodic boundary condition is satisfied (eq. (S15)):

$$\theta(t_0) = \boldsymbol{\theta}(t_0 + \lambda) \tag{S15}$$

We define a function, *F,* that gives the output $\boldsymbol{\theta}(t_0 + \lambda)$ after one oscillation wavelength, λ, for the initial condition, $\boldsymbol{\theta}(t_0)$, such that (eq. (S16)):

$$\theta(t_0 + \lambda) = F\left(\boldsymbol{\theta}(t_0)\right) \tag{S16}$$

After substitution of eq. (S16) into eq. (S15), our periodic boundary condition becomes (eq. (S17)):

$$\theta = F(\boldsymbol{\theta}) \tag{S17}$$

Thus, to satisfy the periodic boundary condition, we need to find the fractional coverages vector, $\boldsymbol{\theta}$, that outputs the same vector $\boldsymbol{\theta}$ after forward integration of one wavelength (function *F*). One method for finding this vector is simply by forward integration until a dynamic steady-state is reached, where we guess a vector $\boldsymbol{\theta}^k$, and define $\boldsymbol{\theta}^{k+1} = F(\boldsymbol{\theta}^k)$ where $\boldsymbol{\theta}^{k+1}$ is the next guess, and iterate until $\boldsymbol{\theta}^{k+1} \approx \boldsymbol{\theta}^k$ is sufficiently satisfied. The efficiency of this algorithm decreases with increasing frequency, for which the method requires forward integration of an indeterminately large number of wavelengths before the periodic boundary condition criteria are satisfied. An alternative approach is using the multivariate Newton-Raphson method, which uses the Jacobian, J, to determine the next initial guess. This method involves first defining a function that we wish to minimize. For a periodic boundary condition this can be defined as minimizing the sum of the square differences between the input and the output of function F for each surface species *i* (eq. (S18)):

$$\min \sum_i g_i(\boldsymbol{\theta}^k) = \sum_i \left(\theta_i^k - F_i(\boldsymbol{\theta}^k)\right)^2 \tag{S18}$$

The Jacobian for the vector function $\boldsymbol{g}(\boldsymbol{\theta}^k)$ is given as (eq. (S19)):

$$J = \begin{bmatrix} \frac{\partial g_1}{\partial \theta_1^k} & \cdots & \frac{\partial g_1}{\partial \theta_n^k} \\ \vdots & \ddots & \vdots \\ \frac{\partial g_n}{\partial \theta_1^k} & \cdots & \frac{\partial g_n}{\partial \theta_n^k} \end{bmatrix} \tag{S19}$$

and describes how the function that is being minimized changes with respect to each fractional coverage. The next guess in the Newton-Raphson method is therefore given by:

$$\boldsymbol{\theta}^{k+1} = \boldsymbol{\theta}^k - \boldsymbol{J}^{-1}\boldsymbol{g}(\boldsymbol{\theta}^k) \tag{S20}$$

such that information provided by the Jacobian guides and accelerates the iterative search for the dynamic steady-state coverages. The process is iterated until an arbitrary criterion $\sum_i g_i(\boldsymbol{\theta}^k) < \varepsilon$ is satisfied. This is one of many methods for finding the local minimum of a function, and other methods may have faster convergence to the local minimum; the primary development of the presented methodology is to reformulate the periodic boundary condition as an optimization problem (eq. (S18)), for which many algorithms can be employed to efficiently find the dynamic steady state at high oscillation frequencies.

We demonstrate the computational speed of the Newton-Raphson method for finding the dynamic steady state by considering the reaction network in Scheme S1. This reaction network is nonlinear because step 3 involves the reaction between two species that are changing in time, A^* and B^*, and thus the differential equations are themselves nonlinear. In this example, we consider the oscillation of rate constants as simple square waves between two states $j = 1$ and $j = 2$, with rate constants for each state given in Table S1. The difference between the two kinetic states lies in the affinity of the catalyst to adsorb A and B, where kinetic state 1 adsorbs B and ejects A^* off the surface, while kinetic state 2 does the opposite.

The convergence of the Newton-Raphson and forward integration methods to the limit cycles are compared in Figure S1 for a frequency $f = 10^2$ Hz. In 11 iteration steps and 1.45 seconds, the Newton-Raphson method converges to the limit cycle value of the fractional coverage of A* at the start of the square wave, $\theta_{\mathrm{A}^*,0}$. Forward integration requires more than 100,000 iterations to reach the same value and takes over 2,000 seconds. The computation times of the two methods are compared across decades of oscillation frequency in Figure S1b. At low frequencies, forward integrations will converge to limit cycles in as little as one oscillation, and thus can be faster than the Newton-Raphson method, which requires the numerical calculation of the Jacobian and may take smaller steps in the low frequency regime. At increasing frequencies, the Newton-Raphson method becomes faster because each integration is over a shorter length of time, while the forward integration method generally becomes slower because more oscillations are required before converging to the limit cycle. The decrease in computation time for the forward integration method at 10^2 Hz is a consequence of changing chemical dynamics, which decreases the total time required before converging to a limit cycle.

Scheme S1. Non-linear reaction network, related to Figure 3.

$\mathrm{A} + * \rightleftarrows \mathrm{A}^*$	k_1, k_{-1}
$\mathrm{B} + * \rightleftarrows \mathrm{B}^*$	k_2, k_{-2}
$\mathrm{A}^* + \mathrm{B}^* \rightleftarrows \mathrm{C}^* + *$	k_3, k_{-3}
$\mathrm{C}^* \rightleftarrows \mathrm{C} + *$	k_4, k_{-4}
Overall: $\mathrm{A} + \mathrm{B} \Rightarrow \mathrm{C}$	

Table S1. The hypothetical rate constants for two kinetic states for the reaction sequence in Scheme S1, related to Figure 3.

	$j = 1$	$j = 2$		$j = 1$	$j = 2$
$k_1^{[j]}$	10^{-5}	10^{5}	$k_{-1}^{[j]}$	10^{-5}	10^{1}
$k_2^{[j]}$	10^{5}	10^{-5}	$k_{-2}^{[j]}$	10^{1}	10^{-5}
$k_3^{[j]}$	10^{1}	10^{1}	$k_{-3}^{[j]}$	10^{-1}	10^{-1}
$k_4^{[j]}$	10^{3}	10^{3}	$k_{-4}^{[j]}$	10^{-1}	10^{-1}

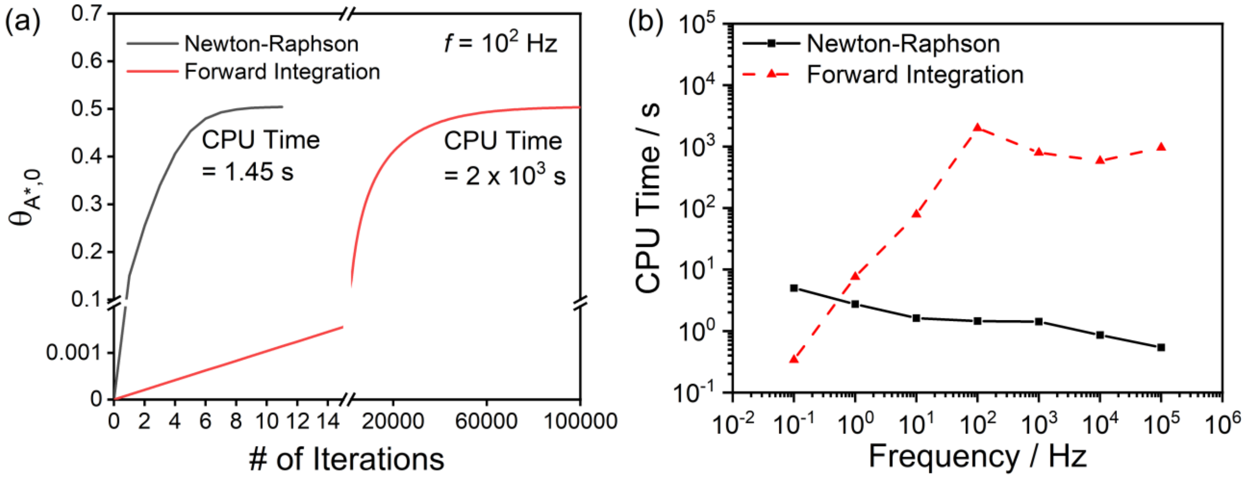

Figure S1. A comparison of iterations and computational time for Newton-Raphson and forward integration methods for the reaction in Scheme S1, related to Figure 3. (a) Comparison of the convergence of the Newton-Raphson method to forward integration using relative and absolute tolerances of 10^{-12} for the built-in Matlab ® solver ode23s. (b) Computation time comparison for Newton-Raphson and forward integration as a function of the frequency. Limit cycle solutions were calculated from an initial guess of $\theta_* = 1$. Related to Figure 3.

For nonlinear reaction systems, such as the network shown in Scheme S1 it is unclear whether one or multiple solutions exist for the periodic boundary value problem. Using a mixture of the Newton-Raphson method and forward integration, the fractional coverage of A* at the periodic boundary, $\theta_{\mathrm{A}^*,0}$, was found as a function of the square-wave oscillation frequency, as shown in Figure S2. At the limits of low and high frequencies, there was only one limit-cycle solution. However, at intermediate frequencies of 10^{-1} to 10^{2} Hz, three limit-cycle solutions were found, one of which was unstable and diverges with any slight perturbation. These unstable limit cycles require the Newton-Raphson solver, because unstable solutions are located at saddle points that locally minimize the criterion in eq. (S18), but can fundamentally never be reached by forward integration. The fractional coverage of A* in the stable (solid) and unstable (dashed) limit cycles are shown in Figure S3; at all conditions, the fractional coverages of θ_* and θ_{C^*} are near zero, and thus the fractional coverage $\theta_{\mathrm{B}^*}(t) \approx 1 - \theta_{\mathrm{A}^*}(t)$.

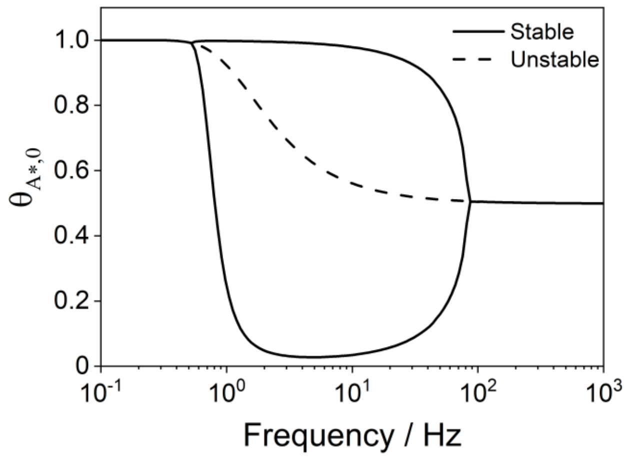

Figure S2. The fractional coverage of A* for limit cycle solutions at the start of a square-wave oscillation (the beginning of kinetic state *j* = 1) for the reaction sequence and kinetics in Scheme S1 and Table S1, respectively. The solid lines are stable solutions and the dashed line is the unstable solution. Related to section, related to Figure 6.

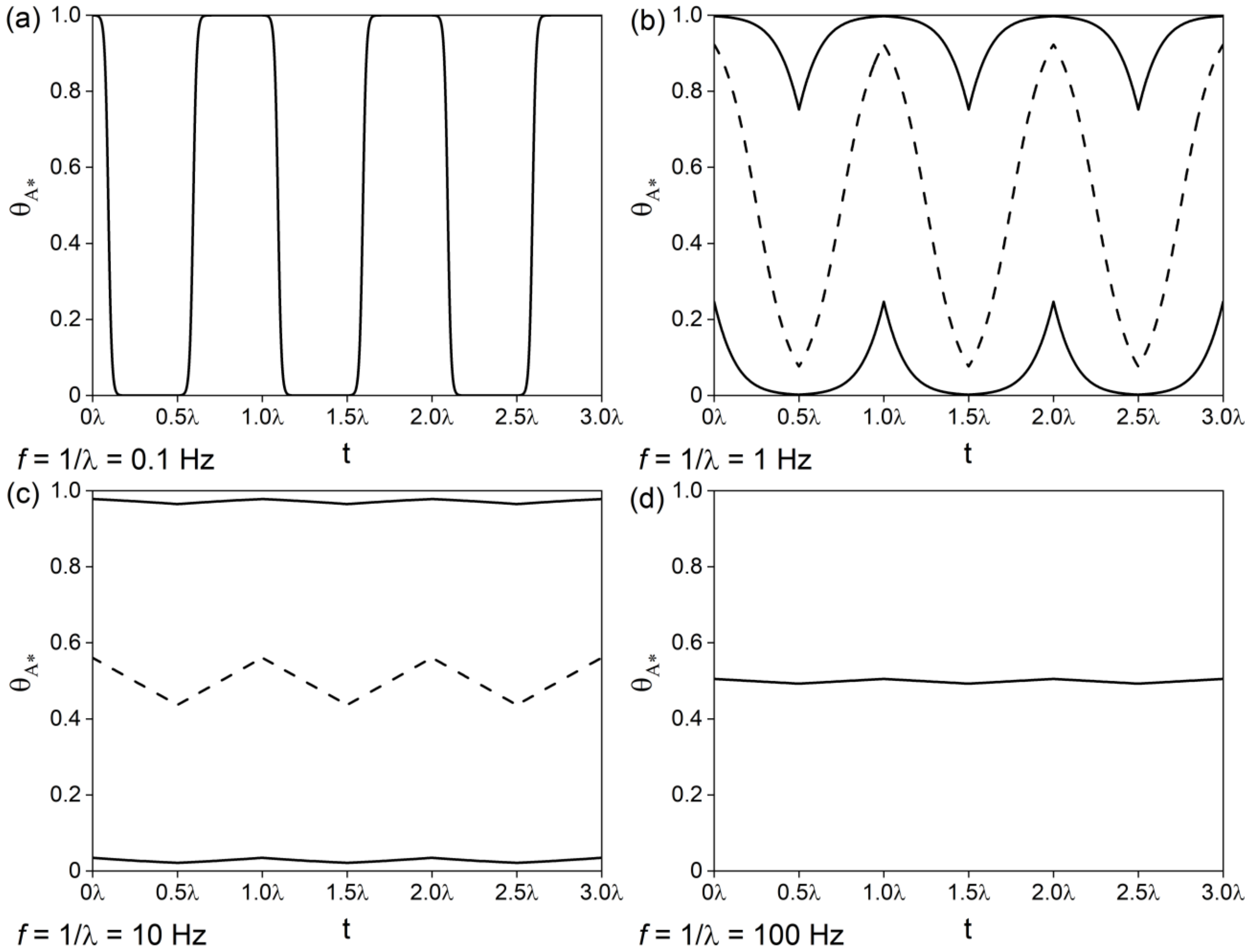

Figure S3. The limit cycles for the fracrtional coverage of A* at varying oscillation frequency for the reaction in Scheme S1 with squarewave oscillation kinetics between states *j* = 1 and *j* = 2 in Table S1. Solid lines are stable limit cycles. Dashed lines are unstable limit cycles. Only limit cycles that satisfy the periodic boundary condition $\boldsymbol{\theta}(t=0) = \boldsymbol{\theta}(t=\lambda)$ were considered. (a) 0.1 Hz, (b) 1 Hz, (c) 10 Hz, and (d) 100 Hz. Related to Figure 6.

In Figure S2, the limiting behaviors at high and low frequencies are connected smoothly by the unstable states, while the stable states diverge sharply at the onset of instability. The unstable states have the property that $\theta_{B^*}(t) \approx 1 - \theta_{A^*}(t) \approx \theta_{A^*}(t - 1/2\lambda)$, and thus the fractional coverages θ_{A^*} and θ_{B^*} oscillate symmetrically about ~0.5. This behavior is stable at the limit of low and high frequencies but becomes unstable at intermediate frequencies. At low frequencies, the oscillation frequency is sufficiently small that the catalyst surface essentially reaches static steady-state in each oscillation, reaching the bounds of $\theta_{A^*} \approx 0$ and $\theta_{A^*} \approx 1$ (Figure S3a). As the frequency increases, the fractional coverages no longer proceed via a sequence of steady states, and the stable solutions diverge at the expense of an unstable limit cycle. At sufficiently large frequencies, the stable solutions separate from the bounds at $\theta_{A^*} \approx 0$ and $\theta_{A^*} \approx 1$ and ultimately converge at the quasi-static surface coverage $\theta_{A^*}(t) \approx 0.5$..

The existence of multiple limit cycles may be problematic in practical application. First, for the reaction in Scheme S1, the stable solutions give surfaces that are much less evenly distributed between A^* and B^*, and thus will have lower rates than the unstable solution. Second, any perturbations in the system may result in jumping from one limit cycle to another, causing unpredictable changes in reaction rate, heat generation, optimal feed composition, and outlet composition—leading to many system controls issues [1]. In practice, regimes of multiple steady states are typically best avoided. In general, for nonlinear reaction systems, we cannot determine the number of possible limit cycles during dynamic catalysis, nor is it clear at what frequencies these multiple limit cycles will arise, though they are likely related to the time scales for kinetic processes (e.g., quasi-equilibrium of reaction or quasi-steady-state of species). This problem has

many similarities to Hilbert's sixteenth problem, as yet unsolved, which concerns the number of limit cycles that exist for a coupled system of two variables with time-independent polynomial differential equations [2]. We can also make no justifiable comment on when solutions with different periodicities or aperiodic, chaotic solutions generally exist under dynamic catalysis conditions; however, we contend that, at the limit of low and high frequencies, there will always be one unique limit cycle solution if the reaction network gives only one *static* steady-state solution, as we discuss next.

At the low frequency limit, if the reaction network allows for only one steady-state solution under static kinetics, as determined by chemical reaction network theory [3], then there exists only one limit cycle during dynamic kinetics. This conclusion is arrived at by recognizing that for sufficiently low frequencies, sufficient time is spent in each kinetic state such that, for most of the time spent in each state, rates and surface coverages are time-invariant. Thus, at the low frequency limit, the fractional coverages of the surface can be approximated as $\theta_{j^*}(t) \approx \theta_{j^*,SS}(t)$, where $\theta_{j^*,SS}(t)$ is the steady-state fractional coverage for species j^* for the kinetics at time t. In this sense, the reaction simply proceeds via a series of static steady states. Therefore, if the reaction network allows for only one steady-state solution during static catalysis, then at each time t there is only one $\theta_{j^*,SS}(t)$—ensuring that there is only one limit cycle during dynamic catalysis.

Section S5. Calculation times and limit cycles for varying number of steps in sine wave approximation

Figure S4a shows the time-averaged rate as a function of frequency for varying n-stepped square-wave approximations of a sine wave, and Figure S4b shows the calculation time for finding the limit cycle. The n = 200 solutions take 30 ms per frequency and vary closely matches the limit cycle solution for n = 10,000 steps, as illustrated in Figure S5. Thus, we consider 200 steps a sufficiently accurate sine wave approximation for this system. Approximating the sine wave as a 200-stepped square wave significantly reduces the computational time by transforming the stiff couple ODEs into linear ODEs that are solved quickly by matrix manipulations.

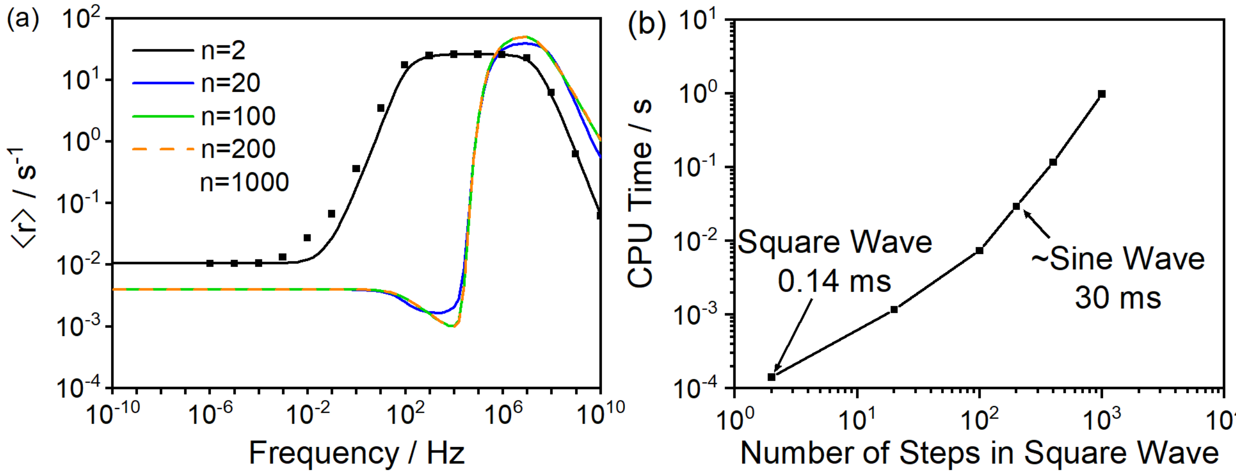

Figure S4. Programmable catalysis rate and computational time for varying number of steps approximating a sinusoidal oscillation. (a) Time-averaged rate as a function of frequency for a sine wave approximated by a stepped-square wave with *n* = 2-1,000 steps. The black squares are the time-averaged rates reported by Ardagh et al. [4] for a square wave with *n* = 2. (b) The median computational time as a function of square wave steps for the time-averaged rate at dynamic steady state for frequencies ranging from 10^{-10} to 10^{10} Hz. A continuous sine wave is well approximated by *n* = 200 steps with a median computation time of 30 ms. Related to Figure 3.

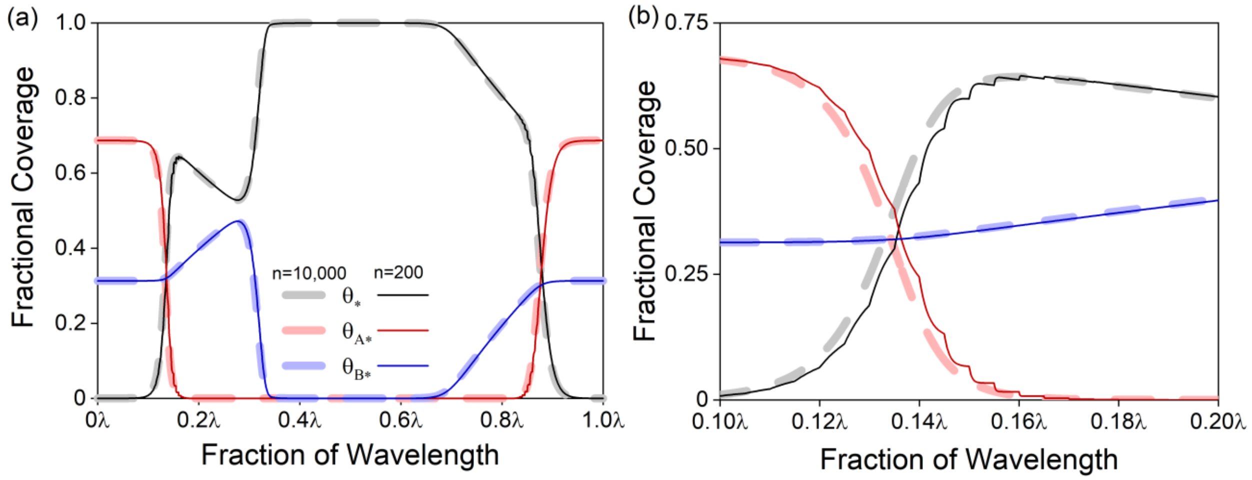

Figure S5. Limit cycle comparison of n = 200 (solid lines) and n = 10,000 steps (dashed lines) when approximating a sinusoidal oscillation. The two solutions nearly overlay. (a) From 0 to 1.0 wavelengths. (b) A closer look at the two solutions from t = 0.1λ to 0.2λ shows the bumps that appear in the n = 200 solution while the n = 10,000 solution is smooth. Related to Figure 3.

Section S6. CO oxidation Matlab codes

Section S6.1. CO oxidation rate constants

This section details the Matlab code for determining the strain and coverage dependent rate constants for CO oxidation on Pt (111). The code is copied below.

```
function k=CO_oxidation_get_rate_constants(strain,T,thetaCO)
%% Steps
% 1 = O2 + * -> O2*
% 2 = O2* + * -> 2O*
% 3 = CO + * -> CO*
% 4 = CO* + O* -> CO2*
% 5 = CO2* -> CO2 + *

%% Import scaling relations
m = [0  0
647.93  60.657
-268.82 78.027
1099.4  217.9
0   0
337.75  175.63
561.77  108.84
-792.68 166.12
-17.232 9.4432
0   0];
dE = m(:,1); E0 = m(:,2);

%% Parameters
MO2 = 32/1000; % kg/mol
MCO = 28/1000; % kg/mol
MCO2 = 44/1000; % kg/mol
NA = 6.02*10^23;
R = 8.314; % J per mol per K
a = 4*(1+strain); % Angstrom
area = sqrt(3)/2*a^2/10^20; % m^2
```

```
%% Rate constants
% Pre-exponential factors
A = ones(1,10)*10^13; A = A'; % s^-1, most are taken to be 10^13

% Collision theory
S1 = 0.05; S3 = 0.84; S_5 = 0.3; % sticking coefficients
A(1) = area*S1*NA./sqrt(2*pi*MO2*R*T)*10^5; % s^-1, factor of 10^5 for Pa to bar
A(5) = area*S3*NA./sqrt(2*pi*MCO*R*T)*10^5; % s^-1, factor of 10^5 for Pa to bar
A(end) = area*S_5*NA./sqrt(2*pi*MCO2*R*T)*10^5; % s^-1, factor of 10^5 for Pa to bar

% Activation Energies
Ea = dE*strain + E0; % kJ/mol
Ea(6)=Ea(6)-96.5*(0.0065*exp(4.79*thetaCO)+0.031*thetaCO*exp(4.79*thetaCO));

% Rate Constants
k = A.*exp(-Ea.*1000./R./T); % s^-1
% k(1) = k1, k(2) = k_-1, k(3) = k2, ...
```

Section S6.2. Calculation of limit cycles

This section details the Matlab code for satisfying the periodic boundary condition for the limit cycle for CO oxidation by using the method of gradient descent. The code is copied below.

```
strain1=-0.05;
strain2=0.05;
T=530;
cycles=10;
x0=[0.2;0.2;0.2;0.2];
counter=1;
for dt=logspace(10,-10,41)
[err,x,inc,ts,xs,rate]=find_limit_cycle(strain1,strain2,T,x0,dt,cycles);
tts{counter}=ts;
xxs{counter}=xs;
rates(counter)=rate;
counter=counter+1;
x0=x;
end

function [err0,x,inc,ts,xs,rate]=find_limit_cycle(strain1,strain2,T,x,dt,cycles)
stepsize=1e-5;inc=0;err0=1e10;gamma=0.1;
flag=0;
while (err0>1e-8 & inc<1e4)
inc=inc+1;
err00=err0;
x
[err0,xs]=fwdsim(strain1,strain2,T,x,dt,cycles);
x00=x;
for j=1:4
   x=x00;
   x(j)=x(j)+stepsize;
   err1(j)=fwdsim(strain1,strain2,T,x+1e-5,dt,cycles);
end
x=x00;
grad=(err1-err0)/1e-5;
if norm(xs-x)>norm(-gamma*grad) & err0<err00
```

```
    xold=x;
    x=xs;
else
    xold=x;
    x=x-gamma*grad;
end
err0
end
[err,xout,t1,x1,t2,x2]=fwdsim(strain1,strain2,T,x+1e-5,dt,cycles);
rate=0;
ks1=CO_oxidation_get_rate_constants(strain1,T,x(4));
ks2=CO_oxidation_get_rate_constants(strain2,T,x(4));
k2=ks1(3);
kr2=ks1(4);
try
rate=rate+trapz(t1,(k2*x1(:,2).*x1(:,1)-kr2*x1(:,3).^2));
k2=ks2(3);
kr2=ks2(4);
rate=rate+trapz(t2,(k2*x2(:,2).*x2(:,1)-kr2*x2(:,3).^2));
rate=rate/(2*dt);
ts=[t1;t2+dt];
xs=[x1;x2];
catch
    rate=0;ts=0;xs=0;
end
end

function [rates]=getrates(strain,T,x)
    x=reshape(x,[],1);
    ks1=CO_oxidation_get_rate_constants(strain,T,x(4));
    P=1.21e-2/1000;%bar
    PO2=0.844*P; %bar
    PCO2=0; %bar
    PCO=0.156*P; %bar
    k1=ks1(1)*PO2;
    kr1=ks1(2);
    k2=ks1(3);
    kr2=ks1(4);
    k3=ks1(5)*PCO;
    kr3=ks1(6);
    k4=ks1(7);
    kr4=ks1(8);
    k5=ks1(9);
    kr5=ks1(10)*PCO2;


    [A11,A12,A10]=getAs(k1,k2,k3,k4,k5,kr1,kr2,kr3,kr4,kr5);
    rates=A11*x+A12*kron(x,x)+A10;
    rnet=k2*x(2)*x(1)-kr2*x(3)^2;
end

function [err,xout,t,x,t2,x2]=fwdsim(strain1,strain2,T,x0,dt,cycles)
x0=reshape(x0,1,[]);
x2=[0*x0;x0];
for j=1:cycles
    [t,x]=ode15s(@(t,x) getrates(strain1,T,x),[0,dt],x2(end,:));
```

```
    [t2,x2]=ode15s(@(t,x) getrates(strain2,T,x),[0,dt],x(end,:));
end
xout=x2(end,:);
err=norm((xout-x0))^2;
end

function [A11,A12,A10]=getAs(k2,k3,k1,k4,k5,kr2,kr3,kr1,kr4,kr5)
%% Steps
% 1 = O2 + * -> O2*
% 2 = O2* + * -> 2O*
% 3 = CO + * -> CO*
% 4 = CO* + O* -> CO2* + *
% 5 = CO2* -> CO2 + *

A10=zeros(4,1);
A11=zeros(4,4);
A12=zeros(4,16);

A10(1)=k5;

A11(1,1)=(- k1 - k2 - k5 - kr4 - kr5); %*
A11(1,2)=(kr2 - k5); %O2*
A11(1,3)=-k5; %O*
A11(1,4)=kr1 - k5; %CO*


A12(1,1)=kr4;   %t0^2
A12(1,2)=(kr4-k3);   %t0*tO2
A12(1,3)=kr4;   %t0*tO
A12(1,4)=kr4;   %t0*tCO
A12(1,5)=0;   %t0*tO2
A12(1,6)=kr3;   %tO2^2
A12(1,7)=0;   %tO*tO2
A12(1,8)=0;   %tCO*tO2
A12(1,9)=0;   %t0*tO
A12(1,10)=0;  %tO*tO2
A12(1,11)=0;  %tO^2
A12(1,12)=k4;  %tCO*tO
A12(1,13)=0;  %t0*tCO
A12(1,14)=0;  %tCO*tO2
A12(1,15)=0;  %tCO*tO
A12(1,16)=0;  %tCO^2

%kr3*tO^2 + k2*t0 - kr2*tO2 - k3*t0*tO2
A11(2,1)=k2; %*
A11(2,2)=-kr2; %O2*
A11(2,3)=0; %O*
A11(2,4)=0; %CO*

A12(2,1)=0;   %t0^2
A12(2,2)=-k3;   %t0*tO2
A12(2,3)=0;   %t0*tO
A12(2,4)=0;   %t0*tCO
A12(2,5)=0;   %t0*tO2
A12(2,6)=0;   %tO2^2
A12(2,7)=0;   %tO*tO2
```

```
A12(2,8)=0;   %tCO*tO2
A12(2,9)=0;   %t0*tO
A12(2,10)=0;  %tO*tO2
A12(2,11)=kr3;  %tO^2
A12(2,12)=0;  %tCO*tO
A12(2,13)=0;  %t0*tCO
A12(2,14)=0;  %tCO*tO2
A12(2,15)=0;  %tCO*tO
A12(2,16)=0;  %tCO^2

%(-kr4)*t0^2 + (2*k3 - kr4)*t0*tO2 + (-kr4)*t0*tO + (-kr4)*t0*tCO + kr4*t0 + (-2*kr3)*tO^2 + (-k4)*tO*tCO
A11(3,1)=kr4; %*
A11(3,2)=0; %O2*
A11(3,3)=0; %O*
A11(3,4)=0; %CO*

A12(3,1)=-kr4;   %t0^2
A12(3,2)=2*k3-kr4;   %t0*tO2
A12(3,3)=-kr4;   %t0*tO
A12(3,4)=-kr4;   %t0*tCO
A12(3,5)=0;   %t0*tO2
A12(3,6)=0;   %tO2^2
A12(3,7)=0;   %tO*tO2
A12(3,8)=0;   %tCO*tO2
A12(3,9)=0;   %t0*tO
A12(3,10)=0;  %tO*tO2
A12(3,11)=-2*kr3;  %tO^2
A12(3,12)=-k4;  %tCO*tO
A12(3,13)=0;  %t0*tCO
A12(3,14)=0;  %tCO*tO2
A12(3,15)=0;  %tCO*tO
A12(3,16)=0;  %tCO^2

%(-kr4)*t0^2 + (-kr4)*t0*tO2 + (-kr4)*t0*tO + (-kr4)*t0*tCO + (k1 + kr4)*t0 + (-k4)*tO*tCO + (-kr1)*tCO
A11(4,1)=k1+kr4; %*
A11(4,2)=0; %O2*
A11(4,3)=0; %O*
A11(4,4)=-kr1; %CO*

A12(4,1)=-kr4;   %t0^2
A12(4,2)=-kr4;   %t0*tO2
A12(4,3)-kr4;   %t0*tO
A12(4,4)=-kr4;   %t0*tCO
A12(4,5)=0;   %t0*tO2
A12(4,6)=0;   %tO2^2
A12(4,7)=0;   %tO*tO2
A12(4,8)=0;   %tCO*tO2
A12(4,9)=0;   %t0*tO
A12(4,10)=0;  %tO*tO2
A12(4,11)=0;  %tO^2
A12(4,12)=-k4;  %tCO*tO
A12(4,13)=0;  %t0*tCO
A12(4,14)=0;  %tCO*tO2
A12(4,15)=0;  %tCO*tO
A12(4,16)=0;  %tCO^2
end
```

Section S7. Calculation of weighted-average rate constants

Through the derivation of eq. (16), we showed that the programmed reaction rate of B formation can be written as a static reaction rate for B* formation containing only rate constants from a single kinetic state. Here, we briefly derive a more general form that applies at all conditions of programmed catalysis and does not rely on reducing the effective reaction network of the chemistry and thus is more broadly applied. In general, for any reaction step, the average rate of an elementary step is given by eq. (S21):

$$\langle r_i \rangle = \frac{\int k_i \theta_j \, dt}{\int dt} \tag{S21}$$

where k_i and θ_j are the relevant rate constant and surface intermediate for the elementary step reaction. We define a parameter, $\bar{k}_i$, that relates the time-averaged coverage, $\langle \theta_j \rangle$, to the time-averaged rate by eq. (S22):

$$\langle r_i \rangle = \bar{k}_i \langle \theta_j \rangle = \bar{k}_i \frac{\int \theta_j \, dt}{\int dt} \tag{S22}$$

Combining eqs. (S21) and (S22) and solving for $\bar{k}_i$ we obtain (eq. (S23)):

$$\bar{k}_i = \frac{\int k_i \theta_j \, dt}{\int \theta_j \, dt} \tag{S23}$$

where we see that $\bar{k}_i$ is a weighted-average rate constant. The utility of this formalism is that average rates of elementary steps $\langle r_i \rangle$ at the limit cycle behave analogously to steady-state rates in static kinetics. For example, in a series reaction, the time-averaged net rate of the overall reaction, $\langle r \rangle$, is given as a difference between the time-averaged forward and reverse reaction rate as (eq. (S24)):

$$\langle r \rangle = \frac{1}{\sigma_i} \left(\langle r_i \rangle - \langle r_{-i} \rangle \right) \tag{S24}$$

where σ_i is the stoichiometric number of elementary step reaction *i*. Finally, the time-average rate of change of surface coverages is given by eq. (S25):

$$\frac{\int \frac{d\theta_j}{dt} dt}{\int dt} = \frac{\int \sum \nu_{ij} r_i \, dt}{\int dt} \tag{S25}$$

where ν_{ij} are the stoichiometric coefficients for each species *j* in each elementary reaction *i*, which is negative for reactants and positive for products. This simplifies to a differential equation of time-averaged quantities in eq. (S26):

$$\frac{d\langle \theta_j \rangle}{dt} = \sum \nu_{ij} \langle r_i \rangle \tag{S26}$$

At a limit cycle, the change of the time-averaged coverage over a period of one oscillation is equal to zero. For the reaction sequence $A + * \leftrightarrow A^* \leftrightarrow B^* \leftrightarrow * + B$ (Scheme S1), this gives eq. (S27) for A* at the limit cycle:

$$\frac{d\langle\theta_{A^*}\rangle}{dt} = 0 = \langle r_1\rangle - \langle r_{-1}\rangle - \langle r_2\rangle - \langle r_{-2}\rangle = \bar{k}_1 a_A\langle\theta_*\rangle - \bar{k}_{-1}\langle\theta_{A^*}\rangle + \bar{k}_2\langle\theta_{A^*}\rangle - \bar{k}_{-2}\langle\theta_{B^*}\rangle \quad \text{(S27)}$$

In general, the system of differential equations at the limit cycle can be written as eq. (S28):

$$\frac{d}{dt}\begin{bmatrix}\langle\theta_*\rangle\\ \langle\theta_{A^*}\rangle\\ \langle\theta_{B^*}\rangle\end{bmatrix} = 0 = \begin{bmatrix}-\bar{k}_1 a_A - \bar{k}_{-3}a_B & \bar{k}_{-1} & \bar{k}_3\\ \bar{k}_1 & -\bar{k}_{-1} - \bar{k}_2 & \bar{k}_{-2}\\ \bar{k}_{-3}a_B & \bar{k}_2 & -\bar{k}_{-2} - \bar{k}_3\end{bmatrix}\begin{bmatrix}\langle\theta_*\rangle\\ \langle\theta_{A^*}\rangle\\ \langle\theta_{B^*}\rangle\end{bmatrix} \quad \text{(S28)}$$

which, when solved, gives the time-averaged coverages of each species at the limit cycle. Equation (S28) is identical to the form that steady-state catalysis takes. Finally, noting the definition of rate in eq. (S24) we obtain a time-average rate function at the limit cycle given in eq. (S29):

$$\langle r\rangle = \frac{\bar{k}_1\bar{k}_2\bar{k}_3 a_A - \bar{k}_{-1}\bar{k}_{-2}\bar{k}_{-3}a_B}{\sum_j\sum_i M_{ij}}$$

$$\boldsymbol{M} = \begin{bmatrix}\bar{k}_2\bar{k}_3 & \bar{k}_{-1}\bar{k}_3 & \bar{k}_{-1}\bar{k}_{-2}\\ \bar{k}_3\bar{k}_1 a_A & \bar{k}_{-2}\bar{k}_1 a_A & \bar{k}_{-2}\bar{k}_{-3}a_B\\ \bar{k}_1\bar{k}_2 a_A & \bar{k}_{-3}\bar{k}_2 a_B & \bar{k}_{-3}\bar{k}_{-1}a_B\end{bmatrix} \quad \text{(S29)}$$

which is identical the rate of a steady-state catalytic reaction except static rate constants k_i are replaced with weighted-average rate constants $\bar{k}_i$. An example calculation of $\bar{k}_2$ for the reaction sequence in Scheme S1 is shown in eq. (S30):.

$$\bar{k}_2 = \frac{k_2^{[1]}\int_0^{\delta t^{[1]}}\theta_{A^*}(t)\,dt + k_2^{[2]}\int_{\delta t^{[1]}}^{\delta t^{[1]}+\delta t^{[2]}}\theta_{A^*}(t)\,dt}{\int_0^{\delta t^{[1]}}\theta_{A^*}(t)\,dt + \int_{\delta t^{[1]}}^{\delta t^{[1]}+\delta t^{[2]}}\theta_{A^*}(t)\,dt} = \frac{k_2^{[1]}\chi_{A^*}^{[1]} + k_2^{[2]}\chi_{A^*}^{[2]}}{\chi_{A^*}^{[1]} + \chi_{A^*}^{[2]}} \quad \text{(S30)}$$

where $\chi_{j^*}^{[n]}$ is defined as the integral of θ_{j^*} over the time of kinetic state [n], and is also equivalent to $\chi_{j^*}^{[n]} = \delta t^{[n]}\langle\theta_{j^*}\rangle^{[n]}$, where $\langle\theta_{j^*}\rangle^{[n]}$ is the time-averaged surface coverage of j* in kinetic state n. The rate constants and $\chi_{j^*}^{[n]}$ values for the reaction conditions in Figure 9 are summarized in Table S2. The corresponding approximate value of $\bar{k}_i$ are given in terms of $k_i^{[j]}$ in Table S3.

The $\bar{k}_i$ in Table S3 are all equal to those in kinetic state [2] except that $\bar{k}_3$ is given by $k_3^{[1]}{\sim}10^{11}\ \mathrm{s}^{-1}$ rather than $k_3^{[2]}{\sim}0.1\ s^{-1}$. The two largest terms in $\boldsymbol{M}$ are $\bar{k}_3\bar{k}_1 a_A$ and $\bar{k}_{-1}\bar{k}_3$, and eq. (S29) simplifies to eq. (S31):

$$\langle r\rangle = \frac{\bar{k}_1\bar{k}_2\bar{k}_3 a_A}{\bar{k}_3\bar{k}_1 a_A + \bar{k}_3\bar{k}_{-1}} = \frac{k_2^{[2]}K_1^{[2]}a_A}{1 + K_1^{[2]}a_A} \quad \text{(S31)}$$

which is identical to what is found in eq. (16). This formulation is more rigorous and will apply to more cases than the methods used to derive eq. (16). The weighted-average $\bar{k}_i$ also describe the effective reversibilities and equilibrium limits, analogous to static rate constants, i.e., eq. (S32):

$$K_{\text{eff}} = \frac{\bar{k}_1 \bar{k}_2 \bar{k}_3}{\bar{k}_{-1} \bar{k}_{-2} \bar{k}_{-3}} \quad \text{(S32)}$$

where $K_{\text{eff}} = a_{\text{B}}/a_{\text{A}}$ is the effective equilibrium constant that is the product-reactant ratio where the time-averaged net rate is zero. Note that $\bar{k}_i$ are not constant but will change as the activity of reactants and products change with extent of reaction. Finally, observe that at the high frequency limit, $\theta_j(t) \to \langle\theta\rangle$, such that $\bar{k}_i \to \langle k_i \rangle$ by eq. (S23). Thus, eq. (S29) simplifies to eq. (14), demonstrating the complete consistency of this formalism.

The failure of the quasi-static mechanism for the example in Figure 9 is that at high frequencies, $\bar{k}_{-1} \to k_{-1}^{[1]} \sim 10^{29}\ \text{s}^{-1}$, which severely limits the sorption of A* when $k_1^{[1]} = 10^6\ \text{s}^{-1}$. The rate approaches that given by eq. (S33). The detrimental increase in $\bar{k}_{-1}$ is not mitigated by the beneficial increase in $\bar{k}_2$ as it approaches $k_2^{[1]}$ because $\omega_{-1} > \omega_2$, giving $\bar{k}_{-1}$ the much more significant enhancement.

$$\langle r \rangle = \frac{\bar{k}_1 \bar{k}_2 a_{\text{A}}}{\bar{k}_{-1}} \approx \frac{k_1^{[1]} k_2^{[1]} a_{\text{A}}}{k_{-1}^{[1]}} \quad \text{(S33)}$$

Table S2. Rate constants and χ parameters, related to Figure 9.

Parameter	[1]	[2]
k_1 (s^{-1})	10^6	10^6
k_2 (s^{-1})	1.8×10^9	52.52
k_3 (s^{-1})	4.46×10^{11}	0.1227
k_{-1} (s^{-1})	1.6×10^{29}	1.2×10^4
k_{-2} (s^{-1})	5.0×10^{-9}	5.3×10^{-4}
k_{-3} (s^{-1})	10^6	10^6
χ_* (s)	10^{-7}	2.15×10^{-8}
χ_{A^*} (s)	5.91×10^{-30}	9.65×10^{-5}
χ_{B^*} (s)	1.07×10^{-13}	3.37×10^{-6}

Table S3. Relationship between weighted-average rate constants and static rate constants, related to Figure 9.

Parameter	Approximate Value	Parameter	Approximate Value
$\bar{k}_1$	$k_1^{[2]}$	$\bar{k}_{-1}$	$k_{-1}^{[2]}$
$\bar{k}_2$	$k_2^{[2]}$	$\bar{k}_{-2}$	$k_{-2}^{[2]}$
$\bar{k}_3$	$k_3^{[1]}$	$\bar{k}_{-3}$	$k_{-3}^{[2]}$

References

[1] Levenspiel, O. (1991). Chemical Reaction Engineering, 3rd ed.; (John Wiley & Sons).

[2] Hilbert, D. (2000). Mathematical Problems. Bulletin of the American Mathematical Society 8, 407–436. 10.1090/S0273-0979-00-00881-8.

[3] Feinberg, M. (1987). Chemical Reaction Network Structure and the Stability of Complex Isothermal Reactors—I. The Deficiency Zero and Deficiency One Theorems. Chemical Engineering Science 42, 2229–2268. 10.1016/0009-2509(87)80099-4.

[4] Ardagh, M. A.; Birol, T.; Zhang, Q.; Abdelrahman, O. A.; Dauenhauer, P. J. (2019) Catalytic Resonance Theory: SuperVolcanoes, Catalytic Molecular Pumps, and Oscillatory Steady State. Catal. Sci. Technol. 9, 5058–5076. 10.1039/c9cy01543d.
